# The Actin Filament-Binding Protein Coronin Regulates Motility in *Plasmodium* Sporozoites

**DOI:** 10.1371/journal.ppat.1005710

**Published:** 2016-07-13

**Authors:** Kartik S. Bane, Simone Lepper, Jessica Kehrer, Julia M. Sattler, Mirko Singer, Miriam Reinig, Dennis Klug, Kirsten Heiss, Jake Baum, Ann-Kristin Mueller, Friedrich Frischknecht

**Affiliations:** 1 Integrative Parasitology, Center for Infectious Diseases, University of Heidelberg Medical School, Heidelberg, Germany; 2 Parasitology, Center for Infectious Diseases, University of Heidelberg Medical School, Heidelberg, Germany; 3 Malva GmbH, Heidelberg, Germany; 4 Department of Life Sciences, Imperial College London, London, United Kingdom; University of Nottingham, UK, UNITED KINGDOM

## Abstract

Parasites causing malaria need to migrate in order to penetrate tissue barriers and enter host cells. Here we show that the actin filament-binding protein coronin regulates gliding motility in *Plasmodium berghei* sporozoites, the highly motile forms of a rodent malaria-causing parasite transmitted by mosquitoes. Parasites lacking coronin show motility defects that impair colonization of the mosquito salivary glands but not migration in the skin, yet result in decreased transmission efficiency. In non-motile sporozoites low calcium concentrations mediate actin-independent coronin localization to the periphery. Engagement of extracellular ligands triggers an intracellular calcium release followed by the actin-dependent relocalization of coronin to the rear and initiation of motility. Mutational analysis and imaging suggest that coronin organizes actin filaments for productive motility. Using coronin-mCherry as a marker for the presence of actin filaments we found that protein kinase A contributes to actin filament disassembly. We finally speculate that calcium and cAMP-mediated signaling regulate a switch from rapid parasite motility to host cell invasion by differentially influencing actin dynamics.

## Author Summary

Parasites causing malaria are transmitted by mosquitoes and need to migrate to cross tissue barriers. The form of the parasite transmitted by the mosquito, the so-called sporozoite, needs motility to enter the salivary glands, to migrate within the skin and to enter into blood capillaries and eventually hepatocytes, where the parasites differentiate into thousands of merozoites that invade red blood cells. Sporozoite motility is based on an actin-myosin motor, as is the case in many other eukaryotic cells. However, most eukaryotic cells move much slower than sporozoites. How these parasites reach their high speed is not clear but current evidence suggests that actin filaments need to be organized by either actin-binding proteins or membrane proteins that link the filaments to an extracellular substrate.

The present study explores the role of the actin filament-binding protein coronin in the motility of sporozoites of the rodent model parasite *Plasmodium berghei*. We found that the deletion of *P*. *berghei* coronin leads to defects in parasite motility and thus lower infection of mosquito salivary glands, which translates into less efficient transmission of the parasites. Our experiments suggest that coronin organizes actin filaments to achieve rapid and directional motility. We also identify two signaling pathways that converge to regulate actin filament dynamics and suggest that they play a role in switching the parasite from its motility mode to a cell invasion mode.

## Introduction

Malaria-causing parasites need to actively migrate at several steps in their complex life cycle [1]. Without motility they would fail, for example, to enter red blood cells or to penetrate the mosquito midgut. The stage with the most formidable motility is the sporozoite, which migrates at average speeds exceeding 1 μm/s through the skin [2,3]. *Plasmodium* sporozoites are formed in parasitic oocysts at the midgut wall of *Anopheles* mosquitoes and, after successful transmission from the mosquito to the mammalian host, ultimately differentiate in hepatocytes to generate red blood cell infecting merozoites. Sporozoites first need to emerge from the oocysts, float through the circulatory fluid of the insect, attach to and actively invade the salivary glands [1]. After ejection with the saliva during the mosquito bite, sporozoites are deposited into the dermis, where they migrate actively at high speed to attach to and enter into blood vessels [1,4]. Taken away with the blood stream they again attach to the liver endothelium and pass through this barrier to finally enter hepatocytes [4,5]. Sporozoites are crescent shaped chiral cells that can also move on diverse substrates without changing their shape at average speeds of 1–2 μm/s [6–8]. The motor driving this gliding motility is located underneath the plasma membrane in a narrow space delimited by a membrane organelle called the inner membrane complex (IMC) that subtends the plasma membrane at a distance of approximately 30 nm. Within this space, it is thought that myosin, anchored in the IMC, drives actin filaments rearwards in what resembles retrograde flow [9,10]. Actin filaments themselves are likely linked to transmembrane proteins that contain adhesive domains including an integrin-like A-domain [9,10]. This linkage thus drives parasite motility upon attachment to a substrate although it is not clear how the different transmembrane proteins transmit force [11–13]. Actin filaments are extremely short in *Plasmodium* as well as in related parasites and cannot be routinely visualized [14–16]. This is at least partly due to a number of differences in the actin monomer structure that prevent the formation of long filaments [17–19]. In addition, actin-binding proteins might play a role in regulating actin filament dynamics. For example, the deletion of actin depolymerizing factor in the related parasite *Toxoplasma gondii* leads to formation of long filaments and stalls motility [15,20]. The *Plasmodium* genome only encodes a small set of canonical actin-binding proteins [21–23]. The only bona-fide actin filament-binding protein in the *Plasmodium* genome is coronin, which shares only 31% sequence identity to *Dictyostelium* coronin [24]. Coronin is conserved among the different species of *Plasmodium* and shows 57% identity between the major human malaria-causing parasite *P*. *falciparum* and the rodent model parasite *P*. *berghei* (S1 Fig).

Coronins are a family of actin filament-binding proteins with the first identified coronin described to be important for cell motility and differentiation in *Dictyostelium discoidum* [25,26]. All coronins have one or two WD40 repeat-containing ß-propellers that mediate actin filament-binding [25,27]. Coronins can harbor 2 independent actin-binding sites in their ß-propellers and can also bind membranes [28,29]. Furthermore, coronins can contain a number of additional domains and regions that allow dimer formation and interaction with a range of different proteins including actin-binding proteins and microtubules [27,30]. In alveolata, a major superphylum of protists, coronins belong to the orphan class of short coronins containing one ß-propeller [27,31,32]. Coronin has been examined in several protozoan parasites such as the human malaria-causing parasite *Plasmodium falciparum*, the related apicomplexan parasite *Toxoplasma gondii* and in *Leishmania*, unicellular parasites from the excavata branch. Yet the coronins in each of these organisms display divergent functions. While in *Leishmania* coronin is essential for microtubule organization, in *Toxoplasma gondii* it only binds actin filaments weakly and plays a minor, uncharacterized role during host cell invasion [31,32]. In *P*. *falciparum* coronin was identified and characterized as an actin filament-binding protein [24] and shown to be able to bind to membranes and bundle actin filaments [33]. These functional differences might be explained by the divergence of coronin among these organisms (S1 Fig).

Here we generated a series of parasite lines that either lack coronin, contain mutations of important residues in the ß-propeller or express coronin fused to mCherry in *P*. *berghei*, a rodent model parasite. These lines show that *P*. *berghei* coronin localizes to the sporozoite periphery in a calcium dependent but actin independent manner and actively contributes to parasite motility through actin filament-binding. During the rapid onset of migration extracellular ligands first trigger intracellular calcium release prior to actin filament formation and coronin relocalization to the rear of the gliding sporozoite. Importantly, coronin is required for efficient transmission of the parasite from mosquitoes to mice as *coronin(-)* parasites and parasites expressing mutated coronin show defects in salivary gland invasion. Lastly, we suggest a switch in signaling pathways linking calcium- and cAMP- mediated signaling during migration and invasion.

## Results

### Deletion of coronin results in aberrant motility on 2-dimensional substrates

To investigate the functions of coronin, we first generated a *P*. *berghei* parasite line lacking the *coronin* gene by transfecting a vector containing a resistance cassette that integrated via double homologous recombination across the coronin open reading frame (S2A Fig). A previous study on *P*. *falciparum* coronin localized the protein in blood stages suggesting a potential role in red blood cell invasion [33]. Remarkably, we readily obtained three different *coronin(-)* parasite lines from two independent transfections suggesting that there was no detrimental effect of the lack of coronin in the blood stage of *P*. *berghei*, where transfection and selection was performed (S2B Fig). Indeed, quantitative analysis of parasite growth in the blood of infected mice revealed no difference to wild type parasites. *Coronin(-)* parasites were transmitted normally to the mosquito vector and resulted in similar oocyst and midgut sporozoite numbers than infection with wild type parasites (Table 1), suggesting that coronin also plays no detectable role in parasite infection of the mosquito midgut. However, we found significantly lower numbers of *coronin(-)* sporozoites accumulating in the salivary glands of mosquitoes (Table 1), with a reduction of the infection rate by 60–70%, suggesting that coronin plays a significant role in invasion of this organ.

**Table 1:** Comparison of the different parasite strains in the infectivity of mosquitoes and the *in vitro* speed of salivary gland sporozoites. Significant differences are indicated with an asterisk. Note that sporozoites from the R24A, R28A mutant, despite lower numbers in the salivary gland moved with comparable speed to the coronin-mCherry parasites. Note that coronin-mCherry parasites are significantly slower than WT parasites.

Parasites	Oocysts (infected midguts)	Ratio Infected vs total midguts	Midgut sporozoites (examined midguts)	Salivary gland sporozoites (examined midguts)	Ratio sal. gland vs midgut sporozoites	Speed in μm/s (examined sporozoites)
**WT**	**54 (51)**	**0.61**	**28000 (55)**	**13000 (75)**	**0.46**	**2.0 (62)**
**Coronin-mCherry**	**58 (40)**	**0.63**	**41000 (50)**	**14000 (79)**	**0.35**	**1.2 (77)**
***coronin(-)***	**72 (44)**	**0.33**	**19000 (202)**	**2900 (247)** *	**0.15** *	**0.2 (85)** *
**Coronin-8mut**	**54 (44)**	**0.50**	**22000 (121)**	**2600 (265)** *	**0.12** *	**0.3 (144)** *
**R24A, R28A**	**63 (57)**	**0.49**	**38000 (53)**	**8200 (262)** *	**0.22** *	**1.2 (56)**
**K283A, D285A**	**76 (78)**	**0.44**	**15000 (153)**	**2700 (322)** *	**0.18** *	**0.3 (61)** *
**R349E, K350E**	**71 (67)**	**0.48**	**20000 (109)**	**4300 (231)** *	**0.22** *	**0.2 (146)** *

* significant differences from the WT for *coronin(-)* and from coronin-mCherry for the four parasite lines expressing mutated coronin-mCherry.

Upon intravenous injection of 10.000 salivary gland-derived sporozoites, the parasitemia of mice receiving *coronin(-)* parasites increased from 0.06% to 1.8% between day 4 to day 6. This was comparable to the increase in mice receiving wild type sporozoites (from 0.14–2.6%). Similarly, during natural transmission by the bites of 10 mosquitoes *coronin(-)* parasites showed an increase from <0.01 to 1.5% parasitemia over the same period, while WT parasites showed an increase from 0.2 to 3.0%, again suggesting no effect of coronin ablation on blood stage growth. However, these data also showed that *coronin(-)* infected mice had consistently lower parasite burdens early during infection. In addition, only 17 of 20 mice were infected with *coronin(-)* parasites after mosquito bites, while all mice bitten by mosquitoes carrying WT sporozoites became infected (Table 2). These, with *coronin(-)*infected mice also showed a slight delay in the onset of blood stage development compared with the wild type controls (Table 2). In contrast, when sporozoites were injected directly into the blood stream of mice there was no difference in the number of infected mice compared to the mosquito bite experiments yet, the onset of a blood stage infection was also slightly delayed (Table 2). The slight delay in blood stage infection went along with fewer animals dying from symptoms of experimental cerebral malaria (ECM), a condition where mice suffer brain hemorrhages early during infection (Table 2). Survival from ECM is often associated with delayed liver stage development [34,35] and was accompanied by a change in the interferon-γ and interleukin-10 levels at the critical time of cerebral malaria symptoms development (S2C Fig). Together these *in vivo* data suggest that *coronin(-)* sporozoites might have a defect in migration in the skin where they are deposited by a mosquito bite and/or during liver stage development. To investigate liver stage growth we infected hepatocytes with wild type and mutant sporozoites and indeed found *coronin(-)* parasites developing slower within liver cells as they form smaller parasites at 48 hours post infection when compared to the wild type (Table 2). We also infected C57BL/6 mice with 10.000 sporozoites and analyzed the parasite load in the liver 42 hours post infection. This showed an overall slightly reduced liver burden in mice infected with *coronin(-)* parasites compared to WT infected mice (S3G Fig).

**Table 2:** Comparison of the different parasite strains for their growth within cultured liver cells and their infectivity for mice. Note that all parasites with a lowered number of sporozoites in their salivary glands (all but WT and coronin-mCherry) failed to infect all mice following mosquito bites. While 100% (24 out of 24) of the mice bitten by mosquitoes infected with either wild type or coronin-mCherry expressing parasites were infected, only 78% (53 out of 68) of the mice bitten by mosquitoes infected with the various mutants developed a blood stage infection. As the motility was similar between WT, coronin-mCherry and the R24A/R28A mutant on one hand and between all the other mutants on the other hand (Table 1), we further analyzed the time to infection of mice successfully infected by mosquitoes. Hence, the first group showed an average time to blood stage infection of 3.4 days and the second group of 3.9 days for i.v. injected parasites (p<0.001, one way ANOVA) and 3.5 versus 4.1 days (p<0.01, one way ANOVA) for mice receiving parasites by mosquito bites.

Parasites	Liver stage size at 24 hpi in μm^2^ (numbers examined)	Liver stage size at 48 hpi in μm^2^ (numbers examined)	Infected mice by i.v. infection / total mice*	Mice suffering from ECM	Infected mice by 10 mosquito bites*	Mice suffering from ECM
**WT**	**39 (30)**	**240 (22)**	**27/27**	**25/27**	**16/16**	**11/16**
			**(3.3)**		**(3.5)**	
**Coronin-**	**41 (28)**	**170 (28)**	**8/8**	**4/8**	**8/8**	**5/8**
**mCherry**			**(3.4)**		**(3.9)**	
**Coronin(-)**	**25 (40)**	**71 (53)** *	**38/38**	**20/38** *	**17/20**	**9/17**
			**(3.8)**		**(4)** *	
**Coronin-**	**34 (32)**	**174 (25)**	**12/12**	**7/12**	**10/12**	**6/10**
**8mut**			**(3.8)**		**(4.1)**	
**R24A,**	**27 (30)**	**191 (34)**	**12/12**	**8/12**	**10/12**	**9/10**
**R28A**			**(3.5)**		**(3.2)**	
**K283A,**	**29 (62)**	**141 (44)**	**12/12**	**5/12**	**5/12**	**3/5**
**D285A**			**(3.9)**		**(3.8)**	
**R349E,**	**31 (35)**	**139 (35)**	**12/12**	**3/12**	**11/12**	**5/11**
**K350E**			**(4)**		**(4.4)**	

*prepatency: time to blood stage infection in parethesis.

*significant differences from the WT for *coronin(-)* and from coronin-mCherry for the 4 parasite lines expressing mutated coronin-mCherry.

**Fig 1:**
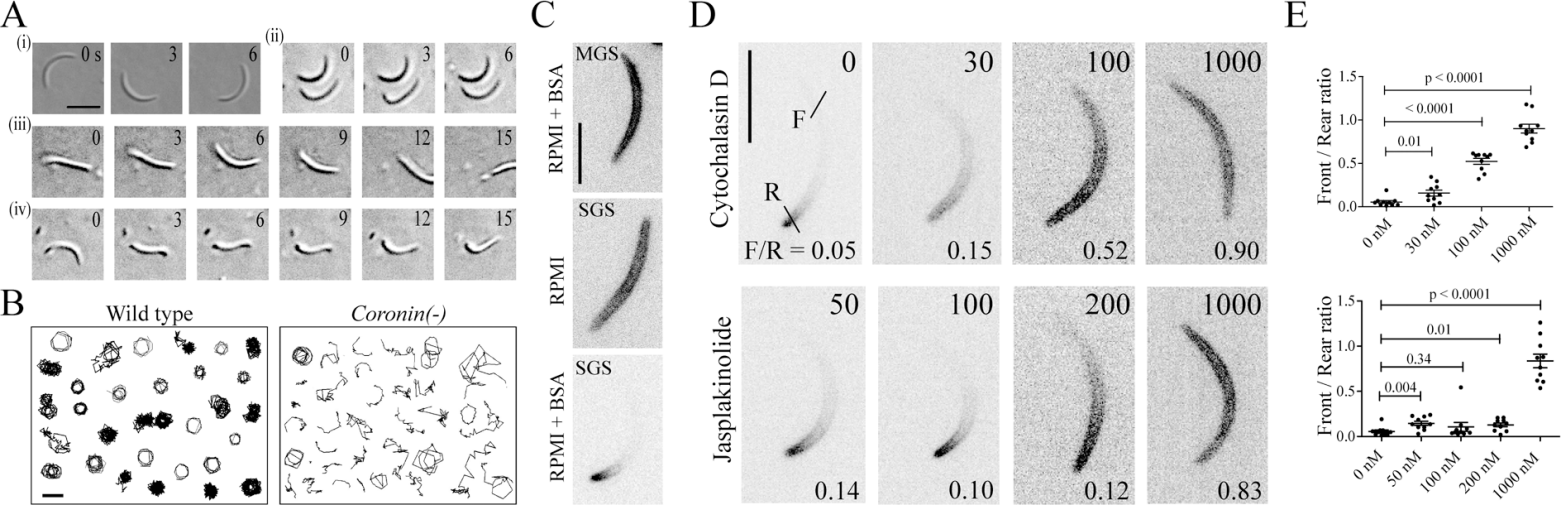
Coronin is important for directional motility and localizes to the rear in an actin-dependent manner. (A) Wild type (WT) *P*. *berghei* strain ANKA parasites (i) and *coronin(-)* (ii-iv) sporozoites were allowed to settle on a glass-bottom Petri-dish and imaged with differential interference contrast microscopy. WT sporozoites move in near-perfect circles with an average speed of about 2 μm/s, while *coronin(-)* sporozoites frequently fail to move or undergo different types of non-persistent movements such as sporadic translocation [37] or various types of flexing behavior (iii-iv). Scale bar: 10 μm. (B) Sporozoites as imaged in A were tracked using ImageJ. Tracks of randomly selected WT sporozoites reveal the persistent circular movement while tracks of *coronin(-)* sporozoites reveal non-circular trajectories. Scale bar: 10 μm. (C) Parasites expressing coronin-mCherry were generated (see S4A Fig) and sporozoites imaged in RPMI medium with a fluorescence microscope in the presence and absence of the motility stimulating bovine serum albumin (BSA). Non-motile sporozoites isolated from the mosquito midgut (MGS) or salivary gland (SGS) showed peripheral fluorescence. Motile salivary gland derived sporozoites showed a fluorescent signal at the rear end. Note the high background in the non-motile sporozoite images indicating the low expression level of coronin-mCherry. There is no quantitative difference in expression level of activated and non-activated sporozoites. Scale bar: 5 μm. (D) Motile sporozoites expressing coronin-mCherry were imaged with a fluorescence microscope under various concentrations of the actin dynamics modulating compounds cytochalasin D and jasplakinolide as indicated at the top right of each panel (in nM). Note the relocalization of the fluorescence signal with increasing drug concentrations. Bars in the top left panel indicate lines where fluorescent intensity profiles were taken to determine front versus rear (F/R) intensity ratios of coronin-mCherry. A low F/R ratio indicates localization to the rear end. Scale bar: 5 μm. (E) Quantitative assessment of the F/R ratio from at least 10 images taken under the indicated concentrations.

To investigate sporozoite motility, we analyzed salivary gland derived *coronin(-)* sporozoites by video microscopy on a 2D glass substrate, where wild type parasites usually move in near-perfect circles (Fig 1A(i)). In contrast to the wild type, *coronin(-)* sporozoites rarely managed to move persistently on circular paths, but often detached from the substrate, frequently moving just back and forth over a single adhesion site (Fig 1A(ii)). This motility form was similar but not identical to patch-gliding [7], which has so far only been observed in sporozoites isolated from the hemolymph. This aberrant motility suggests that the motor or the actin filaments are not properly oriented [7,36]. In addition, *coronin(-)* sporozoites frequently undergo bending and flexing movements without moving forward, a phenotype so far also not described for *Plasmodium* sporozoites (Fig 1Aiii and 1Aiv). As a consequence of these motility defects tracks of the motile *coronin(-)* sporozoites on glass appear different than those of wild type sporozoites (Fig 1B).

### Migration of sporozoites in the skin is independent of coronin

Like with metazoan cells, sporozoite motility defects measured on a flat substrate often do not translate directly into motility defects in a 3-dimensional environment such as the skin [38–40], where sporozoites need to pass through dermal and epithelial skin cells to gain access to the blood [41]. To investigate motility in the skin we thus generated a *coronin(-)* parasite line that strongly expresses mCherry in the sporozoite stage (S3A Fig). These parasites showed the same salivary gland invasion and migration defects as non-fluorescent *coronin(-)* sporozoites (S3B and S3C Fig). To image sporozoites in the skin we let mosquitoes bite the ear of an anesthetized mouse and transferred the mouse to a wide-field fluorescence microscope [3]. Imaging of several bite sites showed that *coronin(-)* sporozoites migrated with similar speed and at similar numbers as did control parasites expressing the same fluorescent proteins (S3D–S3F Fig). This suggests that the main *in vivo* migration defect of coronin ablation is a reduced entry into the salivary gland of mosquitoes and not after ejection into the skin (Tables 1 and 2).

### Coronin localizes to the rear of motile sporozoites in an actin dependent manner

To get further insights into coronin function we next investigated coronin localization in the parasite. To this end we engineered an additional parasite line that expresses a coronin-mCherry fusion protein under the control of the endogenous promoter (S4A and S4B Fig). The mCherry signal could not be visualized in asexual blood stages, gametocytes and ookinetes but was evident in midgut and salivary gland sporozoites. In sporozoites isolated from oocysts and in those isolated from salivary glands, the weak fluorescent signal localized to the periphery of the parasite (Fig 1C). These sporozoites were slightly slower than wild type sporozoites (Table 1). Also the liver stage development and blood stage appearance of coronin-mCherry parasites was somewhat delayed (Table 2) suggesting a minor but measureable impact of the mCherry tag on the function of the fusion protein. During liver stage development coronin continued to be weakly expressed, but the protein appeared cytoplasmic (S4C Fig). Curiously, when bovine serum albumin, which stimulates motility [6,42,43], was added to the salivary gland derived sporozoites, coronin-mCherry relocalized to the rear of the now migrating parasite (Fig 1C and S1 Movie).

To probe if coronin interacts with actin filaments in the sporozoite, we added increasing concentrations of the actin filament modulating compounds cytochalasin D and jasplakinolide to the medium and monitored the distribution of the fusion protein. Both compounds are known to affect sporozoite motility [7,36,44]. To quantify the outcome we measured the intensity profile of the signal across the sporozoite at the front and rear end (Fig 1D) and plotted the difference in maximum fluorescent signal as the front versus rear ratio (Fig 1E). At high concentrations of both compounds coronin-mCherry was no longer localized to the rear end but redistributed to the periphery of the entire parasite (Fig 1D). This showed that already at a low concentration of 30 nM cytochalasin D a relocalization effect was apparent, while for the actin filament stabilizing jasplakinolide this only became apparent above 200 nM (Fig 1E). These observations suggest that coronin is localized to a membrane at the periphery of the parasite, either the plasma membrane or the IMC, and upon activation is moved rearwards with actin filaments.

### Mutant coronins reveal distinct binding to membranes and actin filaments

**Fig 2:**
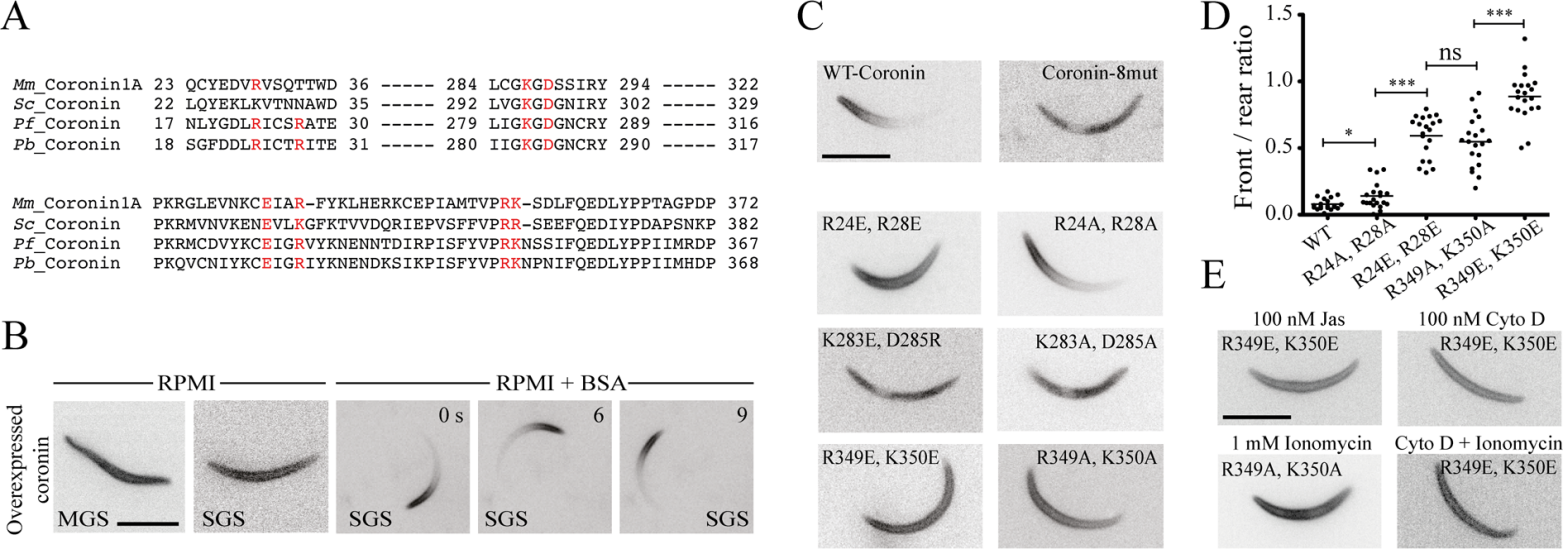
Mutant coronins reveal distinct binding to membranes and actin filaments. (A) Multiple sequence alignment shows that amino acids found to be important for actin binding in yeast are conserved between mouse, yeast and apicomplexans and are marked in red. (B) Fluorescence microscopy of a parasite line overexpressing coronin-mCherry from the uis3 promoter. Note the peripheral fluorescence in non-motile (RPMI) and motile (RPMI+BSA) salivary gland derived sporozoites. Numbers indicate time in seconds. Scale bar: 5 μm. (C) Localization of a number of overexpressed mutant coronin-mCherry fusion proteins. Note the three different types of localizations: cytoplasmic (coronin-8mut; K283A/E, D285A/R), peripheral (R24E, R28E; R349A/E, K350A/E) and polarized (WT, R24A, R28A). Scale bar: 5 μm. (D) Graph showing a quantitative assessment of the front versus rear ratio from 20 images of the various parasite lines overexpressing mutated coronin-mCherry as indicated on the x-axis. (E) The peripheral localization of R349A/E, K350A/E mutants is not altered when sporozoites are incubated with cytochalasin (100 nM), jasplakinolide (100 nM), ionomycin (1000 nM) or cytochalasin D (100 nM) + ionomycin (500 nM) Scale bar: 5 μm.

To further investigate the molecular mechanism of this change in localization and its effect on motility we sought to generate parasite lines expressing mutated versions of coronin. Multiple sequence alignment of both *P*. *berghei and P*. *falciparum* coronin with the mouse and yeast coronin showed conservation of the 4 suggested actin-binding sites (Fig 2A) [45,46]. Due to the low expression of endogenous coronin, we generated another coronin-mCherry parasite line that overexpresses the fusion protein as an additional copy from the stronger sporozoite and liver stage specific *uis3* promoter (S5A and S5B Fig). The coronin-mCherry overexpressed from the *uis3* promoter was detected by western blotting using an antibody against mCherry (S5C Fig). As with the parasite expressing endogenous coronin-mCherry, this parasite line showed clear localization of the protein to the periphery in immature midgut sporozoites and non-activated salivary gland sporozoites, while it relocalized to the rear upon activation (Fig 2B). These coronin-mCherry overexpressing sporozoites were also more motile in medium lacking BSA (18%) compared with the wild type (4%) suggesting that additional coronin leads to stabilization or increased activity of the motility machinery and hence more robust motility.

We next chose to generate a parasite line expressing a mutant coronin-mCherry where 8 conserved residues at four different locations suggested to play a role in actin filament-binding and possible membrane association were replaced by alanines (Fig 2C and S5D Fig). This parasite line showed only cytoplasmic localization of coronin-mCherry (Fig 2C). We next made 6 more parasite lines with three of the amino acid pairs changed into either alanines or amino acids of opposite charge (Fig 2C and S5E Fig). These lines showed remarkable differences in localization. While the R24A, R28A mutant showed a similar localization as the wild type in activated sporozoites, the R24E, R28E mutant localized only to the periphery and did not relocalize to the rear upon activation (Fig 2C). In contrast, the other two mutants showed similar phenotypic localizations independent of their substituted amino acids. Mutations of residues 283 and 285 led to a cytoplasmic localization of the fusion protein, suggesting that these residues are important for membrane association (Fig 2C). Mutation of residues 349 and 350 led to a peripheral localization similar to the R24E, R28E mutant suggesting that these amino acids mediate actin filament-binding as has been suggested for coronins in yeast [45]. A quantitative assessment of the front versus rear ratios further showed that the localization was most disturbed in the R349E, K350E mutant, while the R349A, K350A mutant was similar to R24E, R28E (Fig 2D). If there was however still actin-mediated localization of the mutants to the periphery we anticipated that jasplakinolide or cytochalasin D should lead to a change in localization. This was not the case for either 100 nM or 1 μM of both drugs (Fig 2E), which suggests that indeed coronin is localizing in an actin filament independent fashion to the periphery prior to sporozoite activation. Similarly, activation of secretion by adding ionomycin [43], did not lead to a change in localization of mutant coronin-mCherry in this line (Fig 2E).

### Mutant coronins exhibit defects in sporozoite motility

**Fig 3:**
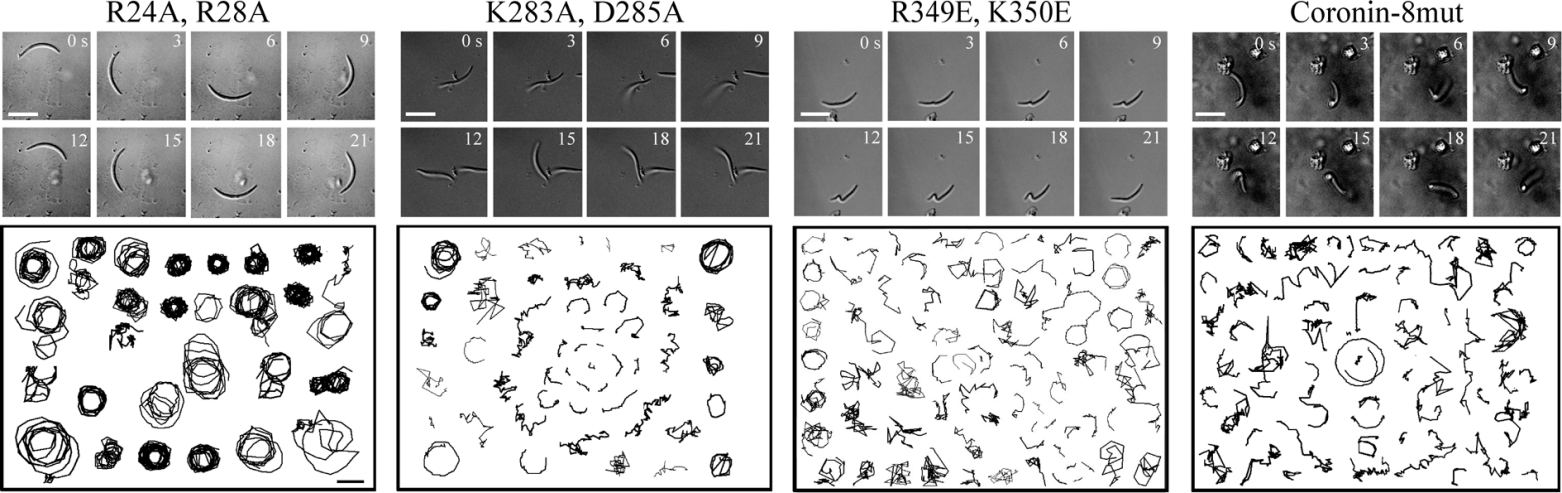
Mutant coronins exhibit defects in sporozoite motility. Time-lapse images and randomly selected tracks of different parasite lines expressing endogenous coronin fused to mCherry with the indicated mutations in their putative actin-binding sites. Note that R24A, R28A moves in a similar manner to WT, while the other mutants move differently and exhibit bending movements so far not seen in any wild type or mutated sporozoite, most strikingly seen in the time lapse of a sporozoites expressing the R349E, K350E mutation. A small but discernable fraction of sporozoite expressing the K283A, D285A mutation in coronin can move in persistent circles. Scale bars: 5 μm (images), 10 μm (tracks).

To investigate the effect of these coronin mutations on motility we generated four additional parasite lines where the endogenous coronin was replaced by four selected mutated forms fused to mCherry (S6 Fig). For these experiments we chose the R24A, R28A mutant, which we expected to behave like wild type coronin, K283A, K285A; R349E, K350E as well as the coronin harboring all 8 amino acid changes, which we expected to show a phenotype different from wild type sporozoites. Like *coronin(-)* parasites, we could not observe any difference in growth of these parasite lines in the blood and early mosquito stages but found different effects on the numbers of sporozoites in the salivary gland, their migration behavior and animal infection capacity (Fig 3, Tables 1 and 2). As expected, the parasite carrying the R24A, R28A mutation showed no difference to wild type control parasites (i.e. endogenous coronin-mCherry expressing parasites). All other parasites however showed severe motility defects similar to those observed in *coronin(-)* sporozoites (Fig 3, Tables 1 and 3).

**Table 3:** Comparison of different parasite lines with different coronin mutants for the percentage of sporozoites showing localization of coronin-mCherry and different movement patterns. The localization is classified as rear, peripheral and cytoplasmic, while the movement pattern is classified as moving, partially moving, attached and drifting.

	WT	*Coronin(-)*	Coronin-	Coronin-	R24A,	K283A,	R349E,
			mCh	8mut	R28A	D285A	K350E
Localization	Rear	—	Rear	Cytoplasm	Rear	Cytoplasm	Periphery
**Moving**	**80**	**4**	**77**	**16**	**74**	**5**	**5**
**Partially moving**	**14**	**35**	**15**	**41**	**13**	**55**	**67**
**Attached**	**3**	**37**	**4**	**33**	**12**	**30**	**22**
**Drifting**	**3**	**23**	**4**	**10**	**1**	**10**	**7**

Of these, the parasite line containing all mutations showed the strongest phenotype. In mosquitoes we found that all but R24A, R28A showed lower numbers of sporozoites in the salivary gland, albeit the ratio of midgut versus salivary gland sporozoites was also low (Table 1). In transmission assays we found that R24A, R28A showed no delay in blood stage appearance although not all animals became infected (Table 2). Of the other mutants only R349E, K350E showed a further delay in blood stage appearance of half a day compared to coronin-mCherry parasites (Table 2). Curiously the size of the liver stages of all mutants was closer to those of wild type parasites as those of *coronin(-)* parasites suggesting that the liver stage phenotype might not be due to the interaction of coronin with actin filaments. Yet, there is a delay of about half a day in the appearance of blood stage parasites of these mutants (Table 2). This hints at a role of coronin prior to hepatocyte invasion, which could be due to a decreased invasion of blood vessels in the skin or decreased extravasation in the liver. Taken together, these data suggest that coronin binds to a peripheral membrane in non-activated sporozoites and following activation binds to actin filaments that move the protein to the rear end. This might also suggest that the role of coronin in sporozoite entry into salivary glands and liver stage development might be due to different mechanisms.

**Fig 4:**
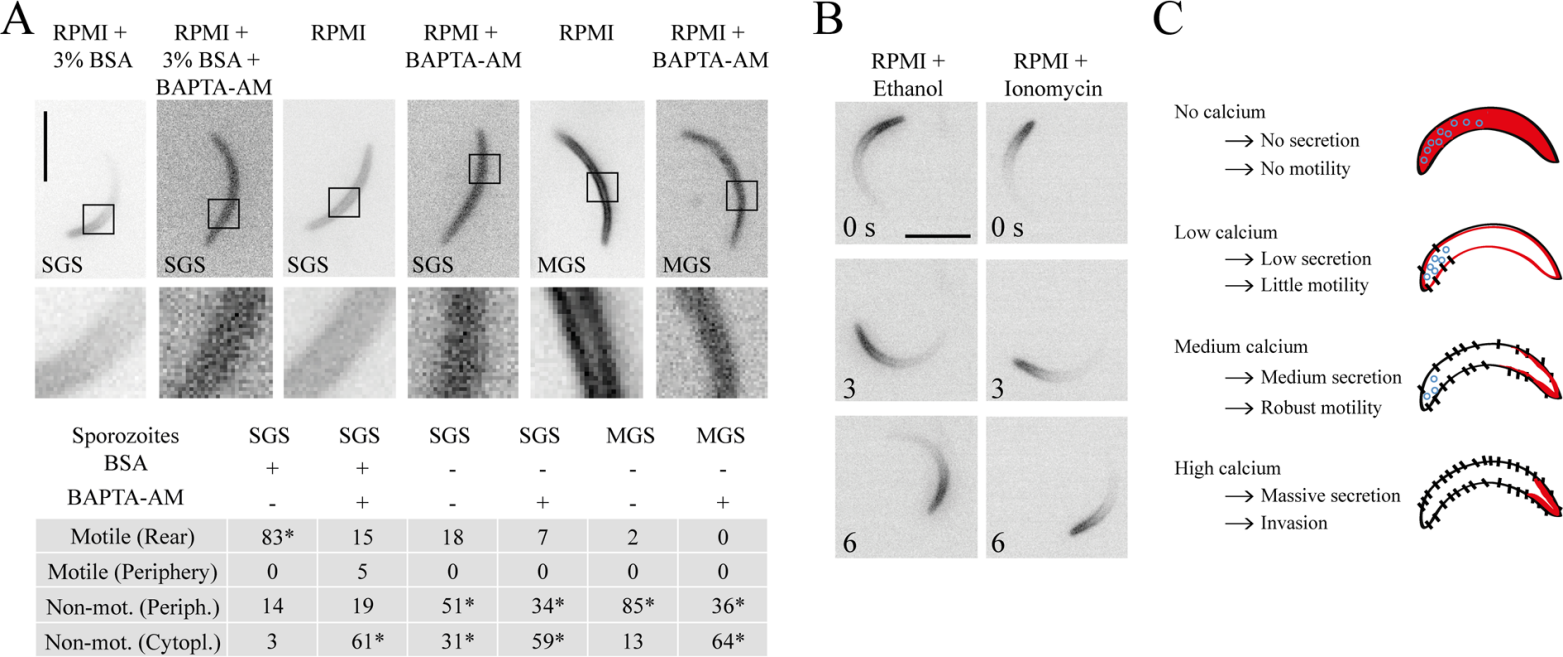
Intracellular calcium release precedes the relocalization of coronin and motility. (A) Activated and non-activated sporozoites overexpressing coronin-mCherry were treated with or without BAPTA-AM to investigate effects on motility and coronin-mCherry localization with fluorescence microscopy. Note that BAPTA-AM ceases motility and relocalizes coronin from the periphery to the cytoplasm in activated salivary gland sporozoites (panel 1–2, top; p < 0.0001). Treatment with BAPTA-AM of non-activated salivary gland sporozoites (panel 3–4, top; p < 0.0002) and midgut sporozoites (panel 5–6, top; p < 0.0001) relocalizes coronin to the cytoplasm. Scale bar: 5 μm. The table shows the percentage of motile and non-motile sporozoites as determined from imaging at least 100 sporozoites and their associated localization pattern of coronin-mCherry [bottom]. The statistical differences are calculated by Fisher’s exact test. (B) Addition of ethanol or of the ionophore ionomycin to non-activated sporozoites leads to coronin-mCherry relocalization to the rear and motility. Scale bar: 5 μm. (C) Model of the role of calcium mediated receptor secretion on sporozoite progression from mosquito to the liver. In the absence of cytosolic calcium coronin (red) localizes to the sporozoite cytoplasm. At low calcium concentrations coronin localizes to the periphery and few TRAP family adhesins are released onto the sporozoite surface. At medium calcium concentrations, coronin localizes to the rear and more adhesins are released onto the sporozoite surface. Signaling associated with these events leads to optimal motility. Finally at very high calcium concentrations massive secretion leads a further increase of adhesins on the surface, which leads to invasion [49].

**Intracellular calcium release precedes the relocalization of coronin and motility** Little is known about the nature of the extracellular activating ligand and the sequence of events leading to sporozoite activation upon ligand binding. Given the different localization patterns of coronin-mCherry in activated versus non-activated sporozoites, we sought to use this parasite line as a visual tool to dissect activating signaling events. Activation of sporozoites with BSA has been shown to include a raise in cytoplasmic calcium [43]. We thus investigated if coronin relocalization appears prior to or after the increase in intracellular calcium following external ligand stimulation. To this end we stimulated sporozoites overexpressing coronin-mCherry with BSA and then chelated intracellular calcium ions with BAPTA-AM. Consistent with previous results [43], BAPTA-AM slowed sporozoites down (Fig 4A). This also resulted in a redistribution of coronin-mCherry to the cytosol (Fig 4A). This redistribution was surprising as we hypothesized that intracellular calcium contributes or leads to actin dependent posterior localization of coronin. We next treated non-activated salivary gland and midgut sporozoites with BAPTA-AM. This also resulted in the cytoplasmic localization of coronin (Fig 4A). We next incubated sporozoites in non-activating medium and added either ionomycin or ethanol, which lead to an increase in intracellular calcium and secretion of vesicles in sporozoites and *T*. *gondii* tachyzoites [47,48]. When treated at low concentrations (100 nM) with these stimulants parasites moved actively for a few minutes with a polarized coronin-mCherry localization (Fig 4B). However, ionomycin treatment did not rescue motility of *coronin(-)* parasites (S7 Fig). This suggests that ligand-dependent calcium elevation appears to be upstream of coronin relocalization but that in the absence of coronin, forced exocytosis does not rescue motility. We also noticed that during treatment with BAPTA-AM, coronin was distributed throughout the cytoplasm. These data suggest that basal levels of intracellular calcium are needed for coronin localization to the periphery in non-activated sporozoites. Moreover, upon ligand stimulation an increase in calcium leads to microneme secretion and coronin relocalization (Fig 4C). Others have shown that increased intracellular calcium also leads to massive exocytosis that mediates invasion in the liver [49] and hence we speculate that different levels of calcium mediate different functions at subsequent stages of the sporozoite journey (Fig 4C).

### cAMP signaling downstream of coronin relocalization modulates motility

**Fig 5:**
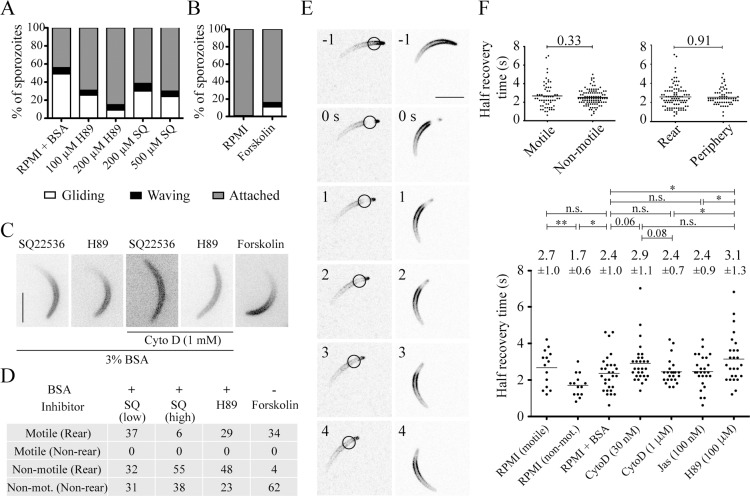
cAMP signaling downstream of coronin relocalization modulates motility. (A) Sporozoites were incubated in RPMI + 3% BSA with the indicated kinase inhibitors at the indicated concentrations, imaged and their movement pattern quantified as gliding, waving or attached. The inhibitors stopped sporozoite motility in a dose-dependent fashion. Over 150 sporozoites were examined for each experiment. Significant differences (Fisher’s exact test) from the controls show both concentrations of H89 tested (p<0.0001) as well as 500 μM SQ22536 (SQ) (p = 0.01; p = 0.46 for 200 μM SQ22536). (B) Forskolin partially stimulates sporozoite motility when added to sporozoites in RPMI. Over 300 sporozoites were examined and their motility pattern quantified as in A. The shown difference is significant (p<0.0001; Fisher’s exact test). (C) Coronin-mCherry expressing sporozoites were investigated under various kinase inhibitors (each at 0,1 mM) with a fluorescence microscope to determine coronin-mCherry localization. The fluorescence stays at the rear in activated sporozoites arrested with PKA inhibitors. Coronin-mCherry relocalizes to the periphery when additional cytochalasin D is applied. Addition of forskolin to non-activated sporozoites leads to the localization of coronin-mCherry to the rear. Scale bar: 5 μm. (D) Table showing percentages of motile and non-motile sporozoites and the associated localization patterns (rear versus non-rear) of coronin-mCherry under the indicated conditions; low and high concentrations of SQ22536 (SQ) were 0,1 and 0,5 mM, respectively. Between 105 and 120 sporozoites were examined per condition. Statistical differences determined by Fisher’s exact test was p<0.0001 from the respective controls listed in the table of Fig 4A. (E) Examples of FRAP of motile sporozoites with coronin-mCherry localized to the rear. Scale bar: 5 μm. Circle indicates location of the bleach spot. (F) Quantitative analysis of FRAP. Coronin-mCherry recovers as fast in motile (>0.25 μm/s) as in non-motile (<0.25 μm/s) sporozoites if pooled across all conditions. There is also no difference in recovery time depending on the localization of coronin-mCherry [top graphs]. Quantitative analysis of FRAP data over a range of conditions [bottom graph]. Average values (+/- S.D.) are indicated above the graph. Bars show significant differences (* p<0.05; ** p<0.01), non-significance (ns) or p-values (Students t-test). Coronin-mCherry recovers significantly faster in non-motile sporozoites incubated in RPMI than in any other condition. With all other conditions there is no difference from each other with the exception of H89, where coronin-mCherry recovers significantly slower when compared to controls, 1 μM Cytochalasin D and 100 nM Jasplakinolide [bottom].

We finally probed a potential involvement of kinases and cAMP signaling in activation of gliding motility using two different cell permeable inhibitors, H89 (inhibits protein kinase A) and SQ22536 (inhibits adenylyl cyclase). All these inhibitors stopped sporozoite motility to some degree as was shown previously [50]. Conversely, when we stimulated cAMP production in the absence of BSA with forskolin, some sporozoites still moved also confirming previous results [50] (Fig 5A and 5B). Remarkably, when sporozoites halted their movement during inhibition by PKA inhibitors, the coronin-mCherry signal remained polarized at the rear (Fig 5C and 5D). This is remarkable as in all our previous experiments the inhibition of motility always altered the localization of coronin from the rear to the periphery (Fig 1D and 1E). This suggests that the inhibition of the cAMP-protein kinase A (PKA) pathway interferes with actin dynamics in a different way to jasplakinolide or cytochalasin D. PKA could phosphorylate coronin and thus lead to altered actin filament-binding characteristics, while the compounds clearly act on actin filaments themselves. To test this, we added cytochalasin D to sporozoites pre-incubated with BSA and H89. This resulted in the peripheral localization of coronin (Fig 5C). Hence, these data suggest that actin filament turnover is halted by PKA inhibition and implies that PKA and coronin are involved in the turnover of actin filaments at the rear. To further probe this we investigated the fluorescence recovery after photo-bleaching (FRAP) of coronin-mCherry overexpressing parasites (Fig 5E). FRAP of motile sporozoites in medium containing BSA recovered a bleached signal within 2.4±0.2 seconds (range: 0.6–4.6 s) to half its original intensity (Fig 5E and 5F). The bleach spot did not move rearwards and there was no directionality in recovery suggesting that the coronin bound actin filaments are stable and that coronin recovers by diffusion (Fig 5E). In medium lacking BSA we bleached motile and non-motile sporozoites. The motile sporozoites recovered within the same time independent of the presence of BSA, while non-motile sporozoites recovered in just 1.7±0.6 seconds. This suggests that binding to actin filaments slows the mobility of coronin-mCherry. Yet, neither cytochalasin D nor jasplakinolide caused a different recovery rate after FRAP compared to controls suggesting that actin filaments play no role in slowing coronin-mCherry mobility (Fig 5F). Curiously, however, incubation with H89 caused a slower recovery (3.1±1.3 s) compared with control parasites and parasites treated with 1 μM cytochalasin D or 100 nM jasplakinolide (Fig 5F). These data suggest a complex level of actin filament regulation by coronin and cAMP mediated signaling that we can currently not resolve. Together, however, these data show a rapid steady-state dissociation of coronin from actin filaments and that coronin mobility is decreased upon sporozoite activation, possibly (but likely only partially) due to its association with actin filaments. The slight increase of recovery under H89 but the absence of an effect of cytochalasin D and jasplakinolide might point to a difference of actin filament dynamics or coronin binding to actin filaments in the presence of these drugs as already suggested by the experiments described in Fig 5C and 5D.

**Fig 6:**
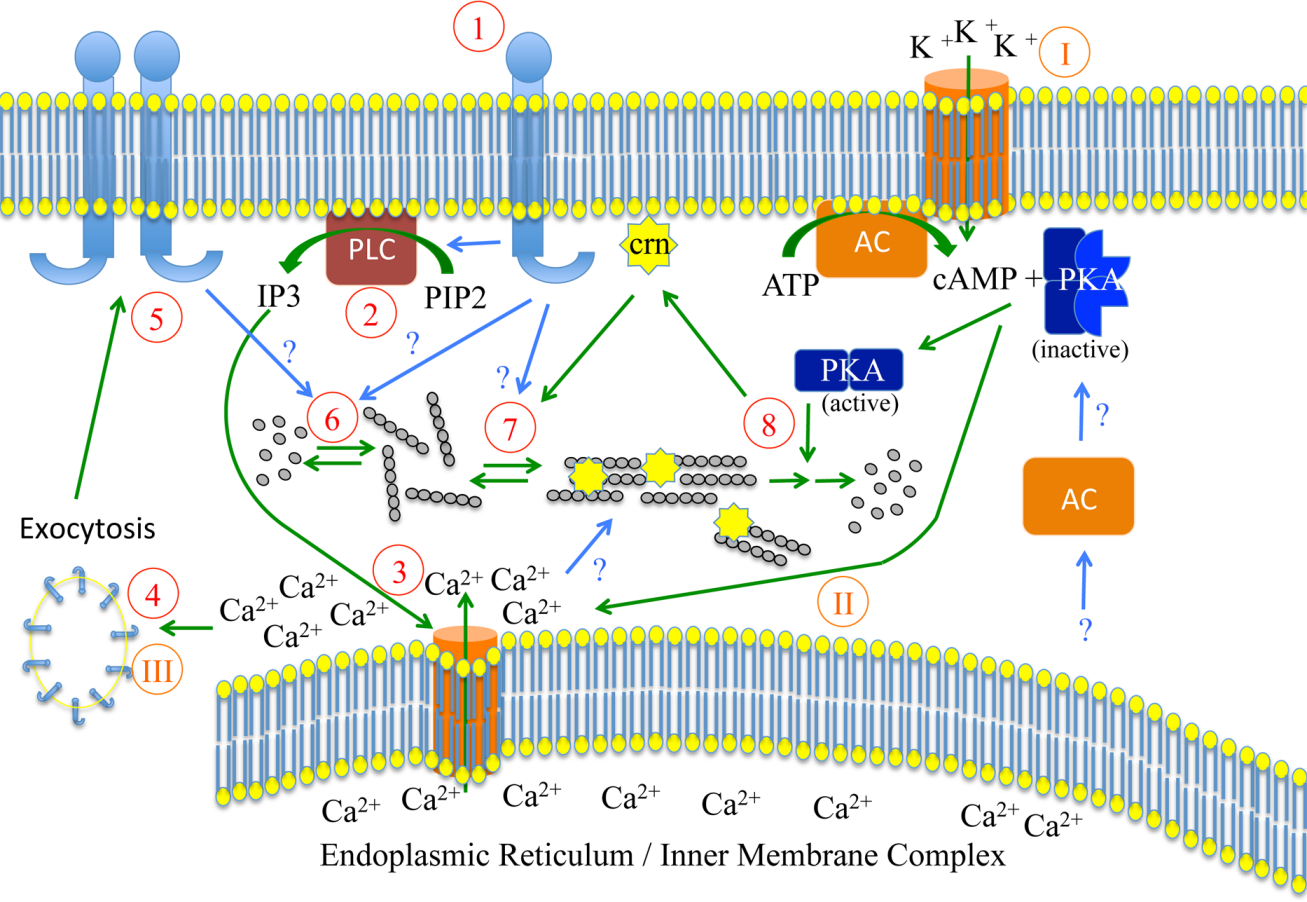
Speculative working model on coronin function at the interface between calcium and cAMP signaling during motility (red Arab numbers) and invasion (orange Roman numbers). Upon activation of a trans-membrane receptor by extracellular ligands on the salivary gland or in the skin (1), phospholipase C is activated and converts PIP2 into IP3 (2). This leads to the release of calcium from intracellular stores (3) and subsequent exocytosis of micronemes, which bring more receptors to the plasma membrane (5). These reinforce signaling and activate actin polymerization (6). Actin filaments are organized by surface receptors and relocalization of coronin from peripheral membranes (either PM or IMC) to actin filaments (7). This leads to efficient adhesion and force production essential for motility in 2D. Coronin (crn) relocalizes from actin filaments as these are disassembled through the action of PKA, which is possibly activated by cytosolic adenylate cyclase (AC). Upon stimulation of membrane bound adenylate cyclase more cAMP is produced leading to higher PKA activity and further calcium release from intracellular stores. The additionally released receptors then mediate invasion.

Taking together, these data and data by other groups [49,51] suggest complex signaling events with feedback loops during activation for gliding. We hypothesize that calcium signaling is upstream of cAMP signaling during gliding, while during invasion these signaling cascades appear reversed (Fig 6).

## Discussion

### Do salivary glands provide a stronger barrier for sporozoites than the skin?

Our data showed that deletion of coronin diminished sporozoite motility and causes a decrease in salivary gland invasion but does not impair gliding within the dermis. While we cannot rule out that sporozoites lacking coronin also have a deficiency in entering the blood circulation, these results suggest that a decrease in gliding affects salivary gland infection more than gliding in the skin. This is in contrast to findings with *hsp20(-)* sporozoites, which also show a severe (but different) decrease in gliding motility but enter salivary glands at wild type levels while moving only very slowly through the skin [7,40]. *hsp20(-)* sporozoites glide very slowly *in vitro* and *in vivo* and appear to attach well to substrates, while *coronin(-)* sporozoites move fast *in vivo* but have problems to stay attached on the surface. Thus it might be that *coronin(-)* sporozoites fail to properly attach to the surface of salivary glands or fail to produce enough force to cross the basal lamina surrounding this tissue, while the 3D environment in the skin allows them to move as efficiently as wild type sporozoites. A similar disconnection of motility in 2D and 3D migration is not uncommon in higher eukaryotic cells [39].

### *Plasmodium* coronin within a wider cell biology picture

None of the coronin isoforms from higher eukaryotes studied so far have been found to be essential for cellular survival. However, several coronins have clearly important functions. For example, coronin assists in organismal survival by mediating proper B- and T-cell functions [52,53]. Coronin function can also be subverted by mycobacteria, which are protected in its presence once engulfed by macrophages [52,53]. In other single-celled eukaryotes such as yeast and *Dictyostelium*, coronin can be ablated without a detrimental effect although it clearly plays a role in actin reorganization in these organisms and might indeed be essential if studied in an environmental rather than tissue culture setting [54,55]. The complex life cycle of *Plasmodium* allowed uncovering of an essential function of coronin in efficient life cycle progression in a rodent model parasite. *P*. *berghei* is frequently used to study mosquito and liver stages instead of the human infecting *P*. *falciparum*. We believe that basic cell biological functions between these two parasites are largely conserved. Along these lines we would expect that coronin will also play a role in sporozoite motility in other Plasmodium species. However, the difference in expression of coronin in blood stages of *P*. *falciparum* versus no expression in *P*. *berghei* is interesting and requires further study. While *P*. *berghei coronin(-)* parasites can be transmitted, the observed decrease in their transmission efficiency to the salivary glands by about two thirds (Table 1) would probably rapidly eliminate *coronin(-)* parasites in an environmental setting. Thus the malaria-causing parasite, in addition to being a medically important cell, provides an intriguing model system for studying subtle effects of proteins on motility in the context of their overall biological role. Such correlations are often missed in higher eukaryotes, where effects such as coronin mediated debranching of actin filaments [56] cannot be correlated with gene deletion studies in mice where no phenotype was observed [53,57]. Here we show that the only bona-fide actin filament-binding protein coronin of *Plasmodium* localizes to the periphery of sporozoites, participates in sporozoite motility (Fig 1) and is essential for efficient transmission of malaria (Table 1).

### Peripheral localization of coronin in sporozoites

At the periphery of sporozoites coronin could localize at the subpellicular network, a cytoskeletal structure underlying the inner membrane complex (IMC), the IMC itself or the plasma membrane. As these structures are only 30 nm apart [8] it is not possible to distinguish them using a light microscope. However, the observation that coronin relocalizes upon sporozoite activation in an actin filament-dependent manner (Figs 1C–1E and 2B) suggests that it is recruited either to the plasma membrane or the IMC. It has been shown that coronin can localize to membranes in macrophages through cholesterol [58] and in neurons via interactions with trans-membrane proteins [59]. Also PIP2 can regulate coronin function at the leading edge of motile cells by inhibiting coronin mediated actin filament disassembly [60]. The N-terminal part of *P*. *falciparum* coronin (i.e. the ß-propeller) was also shown recently to bind to PIP2 [33]. Our work with *P*. *berghei* coronin mutated in the ß-propeller surface revealed that amino acids K283 and D285 might be involved in membrane association. Coronin mutated at these sites did not associate with membranes and parasites expressing a K283A, D285A mutation failed to migrate properly (Figs 2C and 3). This suggests the possibility that coronin could be a linker between the membrane and actin filaments, either directly or through transmembrane proteins, in the same way coronin in neurons can link acetylcholine receptors with the actin cytoskeleton [59]. However, this is clearly too speculative with the current set of data and would require additional biochemical and mutational evidence. We currently prefer a model where coronin is membrane associated in non-activated sporozoites and translocates to actin filaments upon their formation (see below and Fig 6).

### Actin filament-binding by coronin

Actin filament-binding is likely mediated by residues R24, R28, R349 and K350 as mutating these amino acids abrogated relocalization of coronin upon sporozoite activation (Fig 2C and 2D) and mutations in R349 and K350 also lead to aberrant motility (Fig 3). These data also suggest that coronin binds to actin filaments with higher affinity than to membranes. As sporozoites lacking coronin or containing mutations with actin filament-binding defects undergo aberrant motility, this suggests that coronin plays a role in organizing actin filaments that allows for continuous motility. This could be achieved by bundling filaments, if coronin forms dimers as has been shown for other coronins [37,61] including *T*. *gondii* coronin [32]. Alternatively, a single coronin could bundle filaments as observed with the N-terminal half of *P*. *falciparum* coronin [33]. The suggestion of coronin as an actin filament organizer is particularly tempting considering that aberrant movement of the types seen in *coronin(-)* sporozoites can also be observed after adding jasplakinolide or in parasites isolated from the hemolymph [7]. Also parasites lacking the transmembrane protein thrombospondin related anonymous protein as well as parasites lacking the beta subunit of the actin capping protein undergo similar aberrant movement, although these parasites do not enter salivary glands [7,62,63]. Hence a coordination of transmembrane adhesins, actin filaments and actin-binding proteins is clearly needed to achieve continuous motility, which is first needed for salivary gland invasion, a process during which the sporozoites need to cross the basal lamina surrounding the salivary gland. As forced actin polymerization through small doses of jasplakinolide could partially compensate for the loss of the TRAP related adhesin TRAP-like protein (TLP), we previously speculated that actin filament organization is important for motility [11,36]. Jasplakinolide did not rescue the defects of *coronin(-)* sporozoites over a range of concentrations. This suggests that in *tlp(-)* sporozoites actin filaments are organized but the missing link to the substrate causes motility defects that longer filaments can compensate. In contrast, addition of jasplakinolide in *coronin(-)* parasites where actin filaments are disordered would not compensate. Thus, we speculate that coronin is organizing actin filaments such that the stroke of myosins pulls them rearwards, while in *coronin(-)* parasites the filaments are pulled in different directions leading to non-continuous movement (Fig 6). Curiously, this defect is compensated *in vivo*, where *coronin(-)* parasites migrate similarly as wild type parasites in the skin (S3D and S3F Fig). One explanation for this compensation might be that *in vivo* (in 3D) more TRAP-family adhesins link the substrate to the actin filaments than *in vitro* (in 2D).

### Regulation of adhesion formation by coronin

Sporozoite motility is regulated by the formation and turnover of distinct adhesion sites with the substrate [7,40]. Actin filaments and members of the TRAP- family of adhesins also play a role in adhesion formation and turnover [7,11–13]. As parasites lacking coronin frequently detach from the substrate and struggle to re-attach (Fig 1), this also suggests a function for coronin in adhesion formation. Due to the lack of actin filament visibility in sporozoites it remains speculative on how they are organized. Yet, the data collected here further suggest a crucial interplay between TRAP-family adhesins, actin filaments and actin regulating proteins for adhesion formation as a prerequisite for sporozoite migration. The fact that *coronin(-)* sporozoites can migrate at similar rates to wild type parasites in 3D (S3 Fig) further shows that adhesion formation is the limiting factor in motility. This observation has implications for our understanding of how force is transmitted across the plasma membrane in sporozoites and other apicomplexans such as *Toxoplasma gondii*, where most motility studies have been performed in 2D and only recently quantitative studies in 2D and 3D challenge the accepted model for parasite motility [64–66]. It is likely that the loss in motility in 2D is due to a defect in cell adhesion, which can be compensated for in 3D. A similar finding was shown for parasites lacking the TRAP- family adhesin TLP, where *tlp(-)* parasites migrated as well under flow as wild type parasites in the absence of flow [12]. And indeed *tlp(-)* sporozoites are less capable of force generation than WT sporozoites [11].

### Coronin acts downstream of calcium release during sporozoite activation

In macrophages, T- and B-cells coronin is activated during outside-in signaling to further signal for calcium release from intracellular stores [53,67–69]. Our data suggest an opposite signaling pathway. During outside-in signaling, calcium is first released leading to the activation of either coronin itself and/or of the activation of actin filament formation that concomitantly leads to the recruitment of coronin to filaments. Coronin activation could be modulated in different ways, one being through phosphorylation. Indeed coronin was found to be phosphorylated in proteomic screens in *P*. *falciparum* and *T*. *gondii* [70]. One calcium-dependent kinase has already been shown to play a role in parasite motility, although for an earlier stage during mosquito infection [71]. Intriguingly, staurosporine also arrests red blood cell invading parasites in a similar fashion as cytochalasin D [72].

cAMP-mediated signaling plays a role in many motility and invasion processes including in sperm [73]. cAMP signaling also plays a role in invasion of red blood cells by merozoites [51] and of liver cells by sporozoites [49]. When we altered cAMP levels by the application of drugs we found that a number of sporozoites stopped their movement but the coronin signal in these parasites stayed at the rear (Fig 5A–5C). This was in contrast to the relocalization with actin dynamics inhibiting drugs (Fig 1D and 1E). We thus assume that actin filaments are still assembled in parasites treated with the protein kinase A-inhibitor H89 and the adenyl cyclase inhibitor SQ22536. This suggests that cAMP signaling is important for disassembly of actin filaments at the rear, which appears crucial for continuous motility. Also, it suggests that cAMP signaling acts downstream from calcium release, which leads to coronin relocalization. During motility these processes are likely interlinked and they possibly switch during invasion of sporozoites into liver cells, where cAMP signaling was suggested to occur upstream from calcium signaling mediated by a change in the potassium concentration surrounding the sporozoites during transmigration [49] or of pH as in the case of red blood cell invading merozoites [51]. During sporozoite migration we speculate that the two different cAMP signaling pathways initiated by membrane bound adenyl cyclase alpha and cytoplasmic adenyl cyclase beta might be activated differentially during transmigration through cells and extracellular gliding, respectively (Fig 6). Importantly, PKA is likely to phosphorylate coronin as 6 of the 21 phosphorylation sites on coronin show the hallmark of PKA substrates [70,74–76]. Their role could be addressed using the same point-mutagenesis approach as described in Figs 3 and 4.

Taking our results together and placing it within the context of other work [49–51] the following picture emerges for the activation of *Plasmodium* sporozoites (Fig 6): Upon sporozoite contact with the salivary gland basal membrane and again upon transmission into the skin parasites can be activated by a range of ligands from the extracellular matrix or by serum albumin, which signal through a transmembrane protein via phospholipase C to increase intracellular calcium [42,43,50]. This leads to exocytosis of micronemes that bring additional receptors onto the surface leading to increased or sustained signaling. This in turn leads to the polymerization of actin filaments, which are not yet organized. Upon their binding to coronin actin filaments bundle and get translocated by myosin to the rear end, where they accumulate (hence no movement of the bleach spot in Fig 5E) and disassemble to obtain persistent motility (Fig 6). A second signaling pathway through activation of PKA contributes to actin filament disassembly, possibly through coronin, and can be activated by high extracellular potassium ions to switch from a migration to an invasion mode [49]. The two signaling modules in our model appear to be connected such that gliding is occurring when calcium signaling is upstream from cAMP signaling, while invasion occurs upon the switch of these pathways.

## Materials and Methods

### Ethics statement

All animal experiments were performed according to FELASA B and GV-SOLAS standard guidelines. Animal experiments were approved by the German authorities (Regierungspräsidium Karlsruhe, Germany). The project license numbers are G134/14 and G283/14 with a short grant names AAPZ and FAZP, respectively, as assigned by the ethics committee that approved our study.

### Cloning and transfection

Transfection vectors were generated with standard cloning techniques and transfection was performed according to standard protocols [77]. For generation of *coronin(-)* parasites, the vector pB3D+ containing the *Toxoplasma gondii dihydrofolate reductase-thymidylate synthase* (*tgdhfr/ts*) under regulation of the *P*. *berghei* ef1α promoter was used [63]. For generation of mCherry tagged coronin, the vector p262 containing the human dihydrofolate reductase (hDHFR) and the mCherry gene was used. The vector p262 is derived from p236 [78]. The overexpressing mCherry tagged coronin mutants were generated in the B3D+ vector [79]. To generate replacements of the endogenous *coronin* with mCherry tagged *coronin* mutants the previously generated p262-endogenous coronin vector (p262-eCRN) was used. For more details please see supplementary material.

### Mosquito infections

*Anopheles stephensi* mosquitoes (Sda500 strain) were raised at 28°C, 75% humidity, under a 13/11 h light/dark cycle and maintained on 10% sucrose solution containing 0.05% para-aminobenzoic acid. NMRI mice (Charles River) were infected by intraperitoneal injection of 200 μL frozen parasite stocks. After 3 to 5 days post infection the number of exflagellation events were determined with a Zeiss light microscope and a counting grid. If >3 exflagellation events per field of view were observed, mice were anaesthetized with a mixture of 10% ketamine and 2% xylazin in PBS (100 μL per 20 g mouse body weight i.p.) and fed to *Anopheles stephensi* mosquitoes (3–5 days after hatching). Post infection mosquitoes were maintained at 20°C and 80% humidity. Mosquitoes were used 10–23 days post infection for further experiments.

### Analysis of sporozoite motility *in vitro*

Salivary glands of 20–25 mosquitos were dissected in 100 μL RPMI medium without phenol red (GIBCO) in a plastic reaction tube (Eppendorff). Isolated salivary glands were grounded with a pestle and released sporozoites were purified with an Accudenz density gradient [80]. Purified sporozoites were resuspended in 100 μL RPMI containing 3% BSA, transferred to a 96-well plate and centrifuged for 3 min at 1000 rpm. Sporozoites were imaged with differential interference contrast (DIC) and fluorescence using a 25x (NA: 0.8) objective on an inverted light microscope (Axiovert 200M, Zeiss). Video microscopy was performed with taking an image every 3 seconds (unless otherwise stated) for 5 min. When using drugs, we added the drugs after spinning the sporozoites. In some experiments (forskolin and some of the BAPTA-AM experiments), sporozoites were only spun in RPMI without BSA as indicated in the figures. While testing the activation of gliding motility with ionomycin, sporozoites were spun in RPMI medium (R). Ionomycin was added to reach a final concentration of 100 nM without BSA (R + I) or with 3% BSA (R + I + B); [n > 100].

### Analysis of sporozoite motility *in vivo*

Ears of naïve mice were unhaired by treatment with Veet 24 hours prior to intravital imaging to limit autofluorescence. Mosquitos were separated in cups to 10–12 each and starved overnight to increase their biting rate. On the day of imaging, mice were anaesthetized as described above and the ears were placed on the cups containing the infected mosquitoes. Ears were exposed to mosquitoes for 10–15 minutes and formed hematomas were lightly marked with a permanent marker. Bitten mice were placed on the microscope table in such a way that ears could be scanned for hematomas [3]. Upon finding a bite site, sporozoite migration was imaged for 10–15 minutes with an image every three seconds using the Zeiss Axiovert 200M widefield fluorescence microscope.

### Infection of liver cells

HepG2 cells were seeded into 24-well plates with a density of 5x10^4^ and cultivated in DMEM under standard conditions for three days. Salivary glands were isolated 17 days post infection; homogenized with a pestle in 100 μL DMEM and released sporozoites were counted using a Neubauer haemocytometer. 50.000 sporozoites were seeded to each well of a 24-well plate with confluent HepG2 cells. After infection wells were filled up with DMEM to a total volume of 200 μL and incubated for 30 minutes at room temperature. After incubation cells were cultured for further 2 hours under standard cell culture conditions. The medium was removed and wells were washed twice with PBS to remove non-invaded sporozoites. Afterwards, cells were treated with 200 μL (0.05% trypsin) for 10 minutes. To each well 800 μL of DMEM supplemented with Antibiotic-Antimycotic (GIBCO, Life technologies) was added and cells were split in fresh 24-well plates containing cover slips (12x12 mm^2^, thickness 1 mm) for 2 time points. Medium was refreshed every 24 hours.

### Quantification of liver load

4 C57BL/6 mice per parasite line were infected with 10.000 sporozoites by intravenous injection. Livers were removed 42 hours and homogenized in 4 ml of Qiazol (QIAGEN). RNA was isolated from 0.5 ml suspension according to the manufacturer`s protocol. DNase treatment to remove contaminating gDNA was performed using the TURBO DNA-free Kit (AMBION) followed by cDNA synthesis using the First Strand cDNA Synthesis Kit (ThermoFisher). RT-PCR was performed on samples obtained from the cDNA synthesis with and without reverse transcriptase to exclude gDNA contaminations. Quantitative RT-PCR was conducted on a CFX96 Touch Real-Time PCR Detection System (BIO-RAD) with SYBR Green PCR Master Mix (Applied Biosystems). Gene-specific primers for mouse GAPDH and *Plasmodium berghei* 18S rRNA were used.

### Infection of mice

Naive mice were infected by salivary gland sporozoites isolated 17 days post infection either by mosquito bite or by intravenous (i.v.) injection into the tail vein. To infect mice by bite, C57BL/6 mice (*n* = 4 per group per experiment) were anesthetized as described above and individually exposed to cups containing 10–12 mosquitoes. Mosquitoes were starved 24 hours prior to the experiment to increase their biting rate. Mice were exposed to mosquitoes for 10–15 minutes, turning them every 4 minutes. After the experiment blood fed mosquitoes were put on ice and salivary glands isolated to quantify the number of sporozoites. The time between the infection and the first emergence of parasites in the blood, in the following referred to as prepatency, was monitored microscopically by Giemsa stained blood smears. Smears were performed daily from day 3 to day 20 (unless otherwise stated). For intravenous infections, salivary glands of infected mosquitos (17 days post infection) were dissected in 100 μL PBS and homogenized. The number of sporozoites was counted in a Neubauer counting chamber. Sporozoite solutions were diluted with PBS to 10.000 sporozoites per 100 μL and injected i.v. into the tail vein. The prepatency was determined as above. A mouse was considered to be positive if >1 parasite was observed during 5–10 minutes of observations. The survival of infected mice and the appearance of experimental cerebral malaria was monitored for 20 days.

### Cytokine bead array of *coronin(-)* infected mice

30μL of tail blood were taken (every alternate day for 20 days or until mice suffered ECM symptoms) and incubated for 24 hours at room temperature to allow separation of serum and haematocrit. The serum was transferred into new reaction tubes and stored at -20°C until further use. To determine cytokine concentrations 15 μL of serum was analyzed using a multiplex bead array kit (TH1/ TH2/ TH17 BD Biosciences) according to the manufacturer’s protocol. Measurements were done by flow cytometry (BD FACSCalibur) and computational analysis was conducted using FCAP Array Analysis for Mac by Softflow Inc. as described before [35].

### Fluorescence imaging

Imaging was performed on an inverted Zeiss Axiovert 200M microscope using a GFP or rhodamine filter set at room temperature. Images were collected with a CoolSnap HQ2 camera at 1 or 0.5 Hz using Axiovision 4.8 software and 10X (NA: 0.5), 25X (NA: 0.8) or 63X (NA: 1.4) objectives depending on the experimental setting. Image processing was done using ImageJ.

### Fluorescent recovery after photobleaching (FRAP)

For FRAP experiments 2–3 infected salivary glands 17–23 days post mosquito infection were isolated in 100 μL RPMI or RPMI + 3% BSA and grounded with a pestle. Sporozoites expressing mCherry tagged *coronin* were transferred to a glass-bottom culture dish (35 mm Petri dish; 14 mm microwell with No. 1.0 coverglass (MatTek) and mixed with small molecule inhibitors or activators. Imaging was performed on a spinning disc Nikon TE2000 inverted microscope and a 100X (NA: 1.4) objective. The region of interest (ROI) (10x10 pixel size or 1.76 μm^2^) was bleached on each sporozoite followed by imaging for 300 frames with 5 frames per second. The fluorescence intensity of the bleached sporozoite surface was measured using a fixed-size ROI moved by hand to ensure that it is always centered over the moving sporozoites. The fluorescence intensity in a fixed ROI immediately adjacent to the sporozoite was measured and a value for every frame was subtracted from the frapped signal to correct for background fluorescence. As the area imaged was small and sporozoites were moving, it was not possible to correct for photo-damage or sporozoites losing focus. The resulting background-subtracted data was then normalized to the highest intensity of the pre-bleached image. The maximum recovery intensity was determined by averaging the intensities from frame 90 to 140 after bleaching, when recovery was complete for all experiments. The half recovery was calculated as T_1/2_ = (Intensity of Max recovery + Intensity of 1^st^ frame after frap)/ 2 and the time required to reach this intensity determined as half time recovery. Similar results were obtained by fitting the dose response curve in Prism software (GraphPad).

### Protein analysis via western blotting

Sporozoites expressing mCherry tagged *coronin* were isolated from infected salivary glands in RPMI. After centrifugation (7.000 rpm, 5 min) sporozoites were resuspended in RIPA buffer (5 M NaCl, 0.5M EDTA pH 8, 1 M Tris pH 8, 1% NP-40, 10% Na-deoxycholate, 10% SDS, dH_2_O) with protease inhibitors cOmplete, Mini, EDTA-free (Roche) and incubated on ice for 2 hours. 100.000 sporozoites were mixed with loading buffer and loaded onto a Mini-Protean TGX Gel, 4–15% (BioRad). After electrophoresis proteins were transferred onto a Trans-Blot Turbo Mini nitrocellulose membrane using the Trans-Blot Turbo Transfer System (BioRad) followed by blocking of the membrane with 5% milk in Tris (pH 7.5) supplemented with 1% Tween 20 (TBST) for 2 hours at RT. The membrane was probed with the respective primary antibodies diluted in 5% milk in TBST overnight at 4°C, washed 3x with TBST and secondary antibody was incubated in 5% milk in TBST for another hour at room temperature. Proteins were detected using ECL (Pierce). Dilutions of antibody combinations used: rabbit anti-mCherry 1:5.000 (Clontech Laboratories)/ goat anti-rabbit 1:10.000 (GE Healthcare); mouse anti-*Pb*CSP 1:1.000 (Malaria research and reference reagent center: Mr4 mAb3D11)/goat anti-mouse 1:10.000 (GE Healthcare).

## Supporting Information

S1 Fig Comparison of coronins. Schematic showing the domain structure of apicomplexan coronins in comparison to mammalian, yeast and *D*. *discoideum* coronin. Numbers at left indicate percent identities. Numbers on the right indicate length in amino acids.
(TIF)

S2 Fig Analysis of interferon-γ and interleukin-10 concentrations in the blood of mice infected with either WT or *coronin(-)* sporozoites. (A) Schematic of the *coronin* gene deletion strategy using the 5’ and 3’ UTRs of *coronin* to integrate a resistance cassette by double crossover into the *P*. *berghei* strain ANKA locus. Location of primers (S1 File) used for PCR in B are indicated. (B) Diagnostic PCR of 3 independent clones of coronin and the Wild type (WT) control across the entire locus (left) and within the resistance marker as indicated in A. Numbers below the gel pictures show expected amplicon length in base pairs. (C) Graphs showing significantly higher concentration of the cytokine levels at day 10 post infection (red box) in mice infected with *coronin(-)* sporozoites compared to mice infected with WT sporozoites.
(TIF)

S3 Fig Migration of sporozoites in the skin is independent of coronin. (A) Diagnostic PCR to investigate the integration of the *coronin(-)* construct into a fluorescent parasite line [81]. This parasite line expresses mCherry from the CS promoter and GFP from the eF1alpha promoter and showed no difference in growth rates or infectivity across the life cycle when compared with the parental *P*. *berghei* ANKA line. Expected amplicon sizes are indicated below the gel. *coronin(-)*: *coronin* knock out; WL: whole locus; WT: wild type. (B) Table comparing the infectivity of the fluorescent ‘wild type’ and *coronin(-)* lines with the non-fluorescent *P*. *berghei* ANKA wild type and *coronin(-)* lines. Note that the numbers for WT and *coronin(-)* are also printed in Table 1. *denotes significant difference from the control line. (C) *In vitro* time lapse images and tracks of randomly selected sporozoites expressing two fluorescent proteins in the wild type background reveal their persistent circular movement (i), while fluorescent *coronin(-)* sporozoites largely fail to move in a circular fashion [(ii)-(iii)]. Scale bar: 5 μm. (D-G) Analysis of migration in the skin shows no difference in the main parameters between fluorescent WT and *coronin(-)* sporozoites. (D, E) Randomly selected movement tracks from *in vitro*, scale bar: 10 μm (D) and *in vivo*, scale bar: 100 μm (E) experiments. Of 50 WT and 44 *coronin(-)* sporozoites 41 and 37 were moving, respectively. (F) Speed and mean-square displacement of transmitted WT and *coronin(-)* sporozoites migrating in the skin. (G) qRT-PCR analysis of liver burden from mice infected with 10.000 sporozoites. Livers were harvested 42 hours after sporozoites were injected intravenously. ∆CT values from *Pb*ANKA and fluorescent wild type as well as the respective *coronin(-)* infected livers are shown. In addition the sum of all wild type and *coronin(-)* ∆CT values is depicted. Number of mice per parasite line: 4; 3 independent qRT-PCR analyses were performed. ∆CT was calculated by subtracting 18S rRNA CT values from the GAPDH CT values in each sample. P-values from Fischer exact test.
(TIF)

S4 Fig Generation and expression of endogenously tagged coronin-mCherry in liver stages. (A) Schematic of the strategy used for endogenous tagging of *P*. *berghei* coronin. A single crossover-strategy integrated a plasmid containing the resistance cassette (hDHFR) and the *coronin* C-terminal part fused to the coding sequence for mCherry after digestion of the plasmid with BstZ17I. Location of primers (S1 File) used for PCR in B are indicated. (B) Diagnositc PCR to confirm integration of the plasmid. Note that the PCR for the entire locus failed for the integrated plasmid, while a band was readily obtained for the WT locus. (C) Stills showing the expression of endogenous coronin-mCherry in HepG2 cells at 24 hours. and 67 hours post infection. The high background fluorescence in the *coronin*-mCherry channel indicates the relatively low expression level. HSP70 staining was used to identify liver stage parasites and Hoechst to reveal host cell and parasite DNA. Scale bar: 15 μm.
(TIF)

S5 Fig Generation and diagnosic PCRs of the parasite lines overexpressing coronin-mCherry from the *uis3* promoter. (A) Schematic of the strategy used to generate *P*. *berghei* lines expressing coronin-mCherry from the *uis3* promoter. A single crossover-strategy integrated a plasmid containing the resistance cassette (hDHFR) and *coronin* fused to the coding sequence for mCherry after digestion of the plasmid with NdeI within the 5’UTR of *uis3*. Locations of primers (S1 File) used for PCR in B are indicated. (B) Genotyping PCR indicating desired integration of WT-*coronin*-mCherry in the UIS3 locus (5’ int, 3’ int and selection marker). Expected amplicon sizes are indicated below the gel. Band with * is non-specific due to non-clonality of the population. (C) Western blot showing expression of coronin-mCherry in salivary gland sporozoites probed with antibodies against mCherry (lane 1). The antibodies against circumsporozoite protein (CSP) were used as control (lane 3). CSP (lane 4) but not mCherry (lane 2) is detected in WT sporozoites. 10^5^ sporozoites were loaded per lane. (D-E) Genotyping PCR showing proper integration of coronin mutants, Coronin-8mut (D) and R24A, R28A; R24E, R28E; K283A, D285A; K283E, D285R; R349A, K350A; R349E, K350E (E) (bands with * are non-specific due to the non-clonal population). Expected amplicon sizes are indicated below the gels.
(TIF)

S6 Fig Generation and diagnosic PCRs of the mutated coronin-mCherry lines. (A) Double homologous integration strategy for replacement of coronin mutants. Locations of primers (S1 File) used for PCR in B and restriction sites for generation of the linear DNA used for transfection are indicated. (B-C) Genotyping PCR confirming the replacement of endogenous coronin with coronin-8mut (B) and R24A, R28A; K283A, D285A; R349E, K350E (C). Expected amplicon sizes are indicated below the gels.
(TIF)

S7 Fig Forced exocytosis does not rescue the *coronin(-)* motility phenotype. (A) Speed of sporozoites imaged in RPMI (R), RMPI supplemented with 100 nM ionomycin (R+I) and RMPI supplemented with 100 nM ionomycin and 3% bovine serum albumin (R+I+B). Note that while WT parasites move increasingly faster with ionomycin and ionomycin+BSA, there is no increase in motility in *coronin(-)* parasites. (B) Table listing the different movement patterns of WT and *coronin(-)* sporozoites incubated in RPMI (R), RPMI + 100nM ionomycin (R+I) and RPMI + 100nM ionomycin + 3% BSA (R+I+B). Note the increased numbers of motile parasites (red).
(TIF)

S1 File Primers used in this study. (DOCX)

S1 Movie Salivary gland sporozoites activated with serum albumin showing endogenous coronin-mCherry localizes to the rear in motile sporozoite. One image was taken every 3 seconds using a 63x (1.4 NA) objective.
(GIF)
