# Correction: The Actin Filament-Binding Protein Coronin Regulates Motility in *Plasmodium* Sporozoites

**DOI:** 10.1371/journal.ppat.1005818

**Published:** 2016-08-02

**Authors:** The PLOS Pathogens Staff

In Table 2, dividing lines were introduced during typesetting that affect the readability of the table. The publisher apologizes for the error. Please see the corrected Table 2 here.

**Table 2:** Comparison of the different parasite strains for their growth within cultured liver cells and their infectivity for mice.

Parasites	Liver stage size at 24 hpi in μm^2^ (numbers examined)	Liver stage size at 48 hpi in μm^2^ (numbers examined)	Infected mice by i.v. infection / total mice*	Mice suffering from ECM	Infected mice by 10 mosquito bites*	Mice suffering from ECM
**WT**	**39 (30)**	**240 (22)**	**27/27** **(3.3)**	**25/27**	**16/16** **(3.5)**	**11/16**
**Coronin-mCherry**	**41 (28)**	**170 (28)**	**8/8** **(3.4)**	**4/8**	**8/8** **(3.9)**	**5/8**
**Coronin(-)**	**25 (40)**	71 (53)*	**38/38** **(3.8)**	20/38*	**17/20** (4)*	**9/17**
**Coronin-8mut**	**34 (32)**	**174 (25)**	**12/12** **(3.8)**	**7/12**	**10/12** **(4.1)**	**6/10**
**R24A, R28A**	**27 (30)**	**191 (34)**	**12/12** **(3.5)**	**8/12**	**10/12** **(3.2)**	**9/10**
**K283A, D285A**	**29 (62)**	**141 (44)**	**12/12** **(3.9)**	**5/12**	**5/12** **(3.8)**	**3/5**
**R349E, K350E**	**31 (35)**	**139 (35)**	**12/12** **(4)**	**3/12**	**11/12** **(4.4)**	**5/11**

*prepatency: time to blood stage infection in parethesis.

*significant differences from the WT for *coronin(-)* and from coronin-mCherry for the 4 parasite lines expressing mutated coronin-mCherry.
