# Supplementary figures and images for: The Actin Filament-Binding Protein Coronin Regulates Motility in Plasmodium Sporozoites
Source: PLoS Pathog. 2016 Jul 13;12(7):e1005710. doi: 10.1371/journal.ppat.1005710 (PMC4943629; doi:10.1371/journal.ppat.1005710)

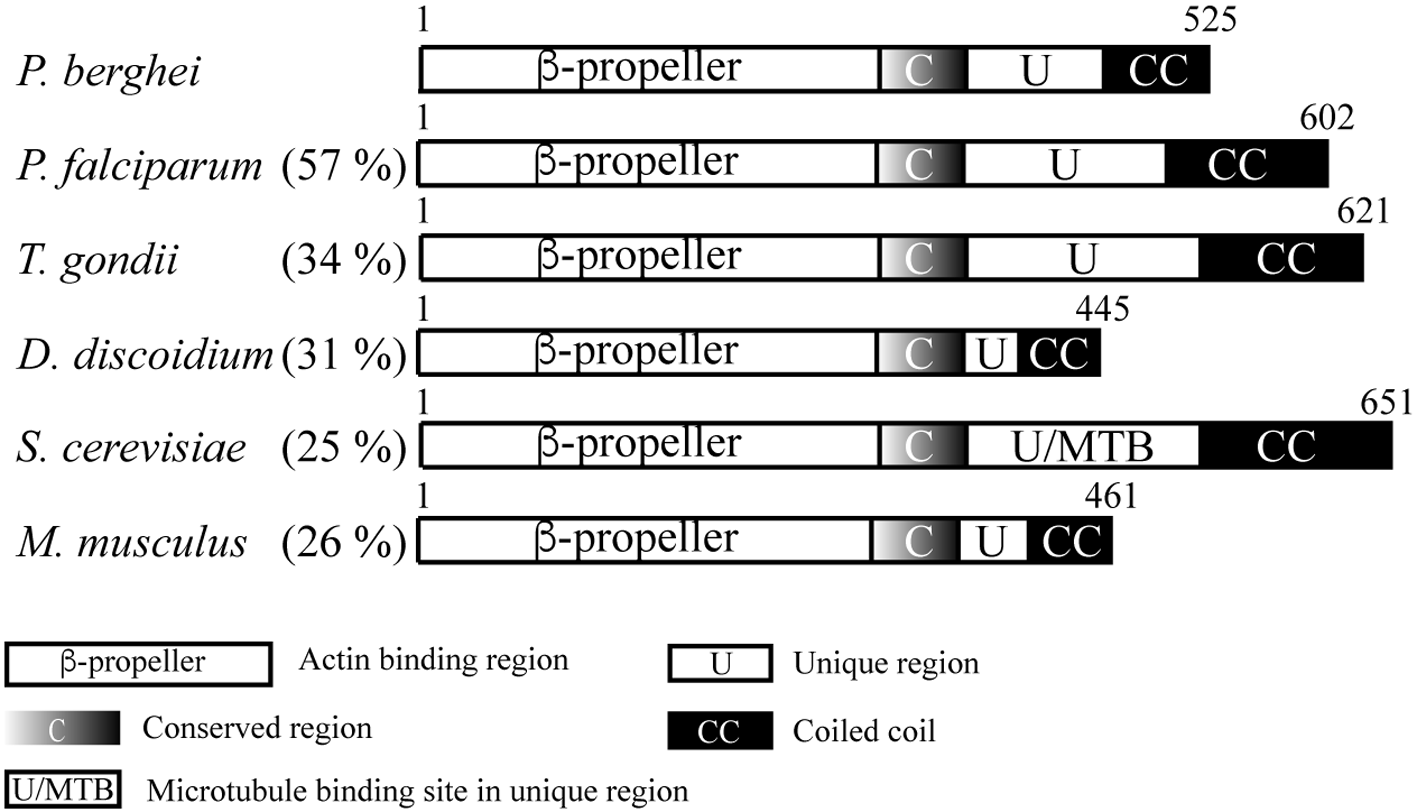

Supplement: S1 Fig — Schematic showing the domain structure of apicomplexan coronins in comparison to mammalian, yeast and D. discoideum coronin. Numbers at left indicate percent identities. Numbers on the right indicate length in amino acids. (TIF) [file ppat.1005710.s001.tif]

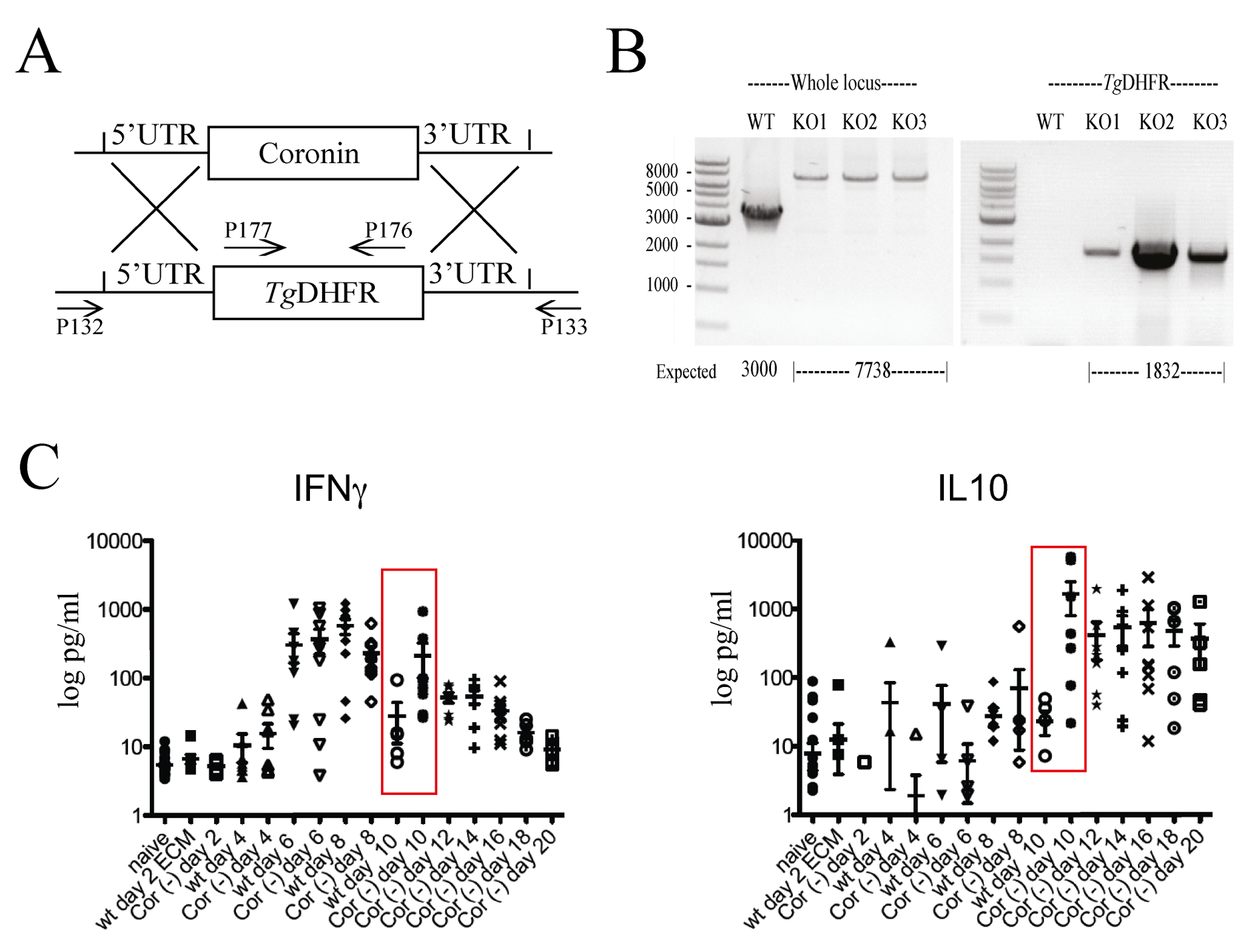

Supplement: S2 Fig — (A) Schematic of the coronin gene deletion strategy using the 5’ and 3’ UTRs of coronin to integrate a resistance cassette by double crossover into the P. berghei strain ANKA locus. Location of primers (S1 File) used for PCR in B are indicated. (B) Diagnostic PCR of 3 independent clones of coronin and the Wild type (WT) control across the entire locus (left) and within the resistance marker as indicated in A. Numbers below the gel pictures show expected amplicon length in base pairs. (C) Graphs showing significantly higher concentration of the cytokine levels at day 10 post infection (red box) in mice infected with coronin(-) sporozoites compared to mice infected with WT sporozoites. (TIF) [file ppat.1005710.s002.tif]

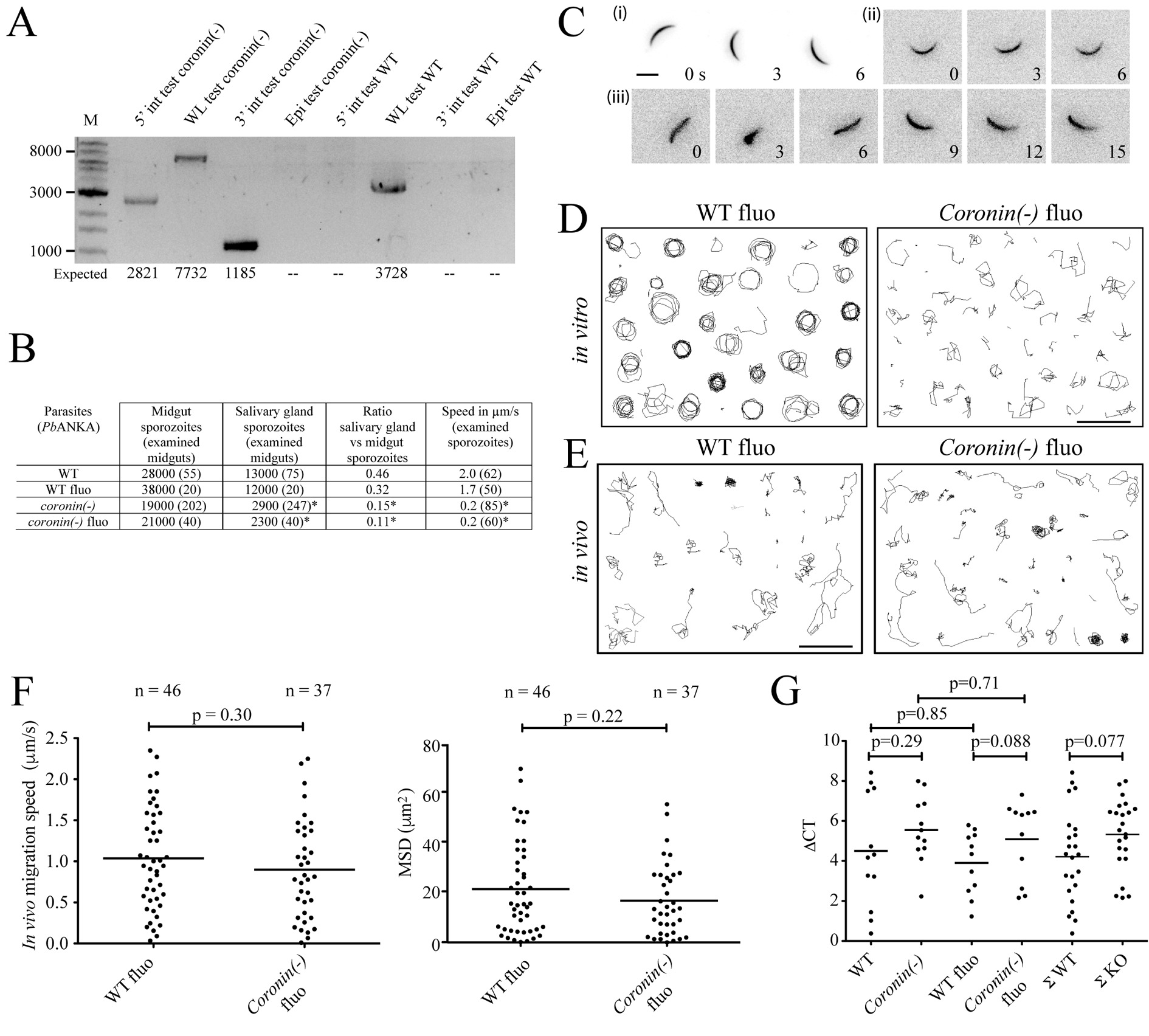

Supplement: S3 Fig — (A) Diagnostic PCR to investigate the integration of the coronin(-) construct into a fluorescent parasite line [81]. This parasite line expresses mCherry from the CS promoter and GFP from the eF1alpha promoter and showed no difference in growth rates or infectivity across the life cycle when compared with the parental P. berghei ANKA line. Expected amplicon sizes are indicated below the gel. coronin(-): coronin knock out; WL: whole locus; WT: wild type. (B) Table comparing the infectivity of the fluorescent ‘wild type’ and coronin(-) lines with the non-fluorescent P. berghei ANKA wild type and coronin(-) lines. Note that the numbers for WT and coronin(-) are also printed in Table 1. *denotes significant difference from the control line. (C) In vitro time lapse images and tracks of randomly selected sporozoites expressing two fluorescent proteins in the wild type background reveal their persistent circular movement (i), while fluorescent coronin(-) sporozoites largely fail to move in a circular fashion [(ii)-(iii)]. Scale bar: 5 μm. (D-G) Analysis of migration in the skin shows no difference in the main parameters between fluorescent WT and coronin(-) sporozoites. (D, E) Randomly selected movement tracks from in vitro, scale bar: 10 μm (D) and in vivo, scale bar: 100 μm (E) experiments. Of 50 WT and 44 coronin(-) sporozoites 41 and 37 were moving, respectively. (F) Speed and mean-square displacement of transmitted WT and coronin(-) sporozoites migrating in the skin. (G) qRT-PCR analysis of liver burden from mice infected with 10.000 sporozoites. Livers were harvested 42 hours after sporozoites were injected intravenously. ∆CT values from PbANKA and fluorescent wild type as well as the respective coronin(-) infected livers are shown. In addition the sum of all wild type and coronin(-) ∆CT values is depicted. Number of mice per parasite line: 4; 3 independent qRT-PCR analyses were performed. ∆CT was calculated by subtracting 18S rRNA CT values from the GAPDH CT values [file ppat.1005710.s003.tif]

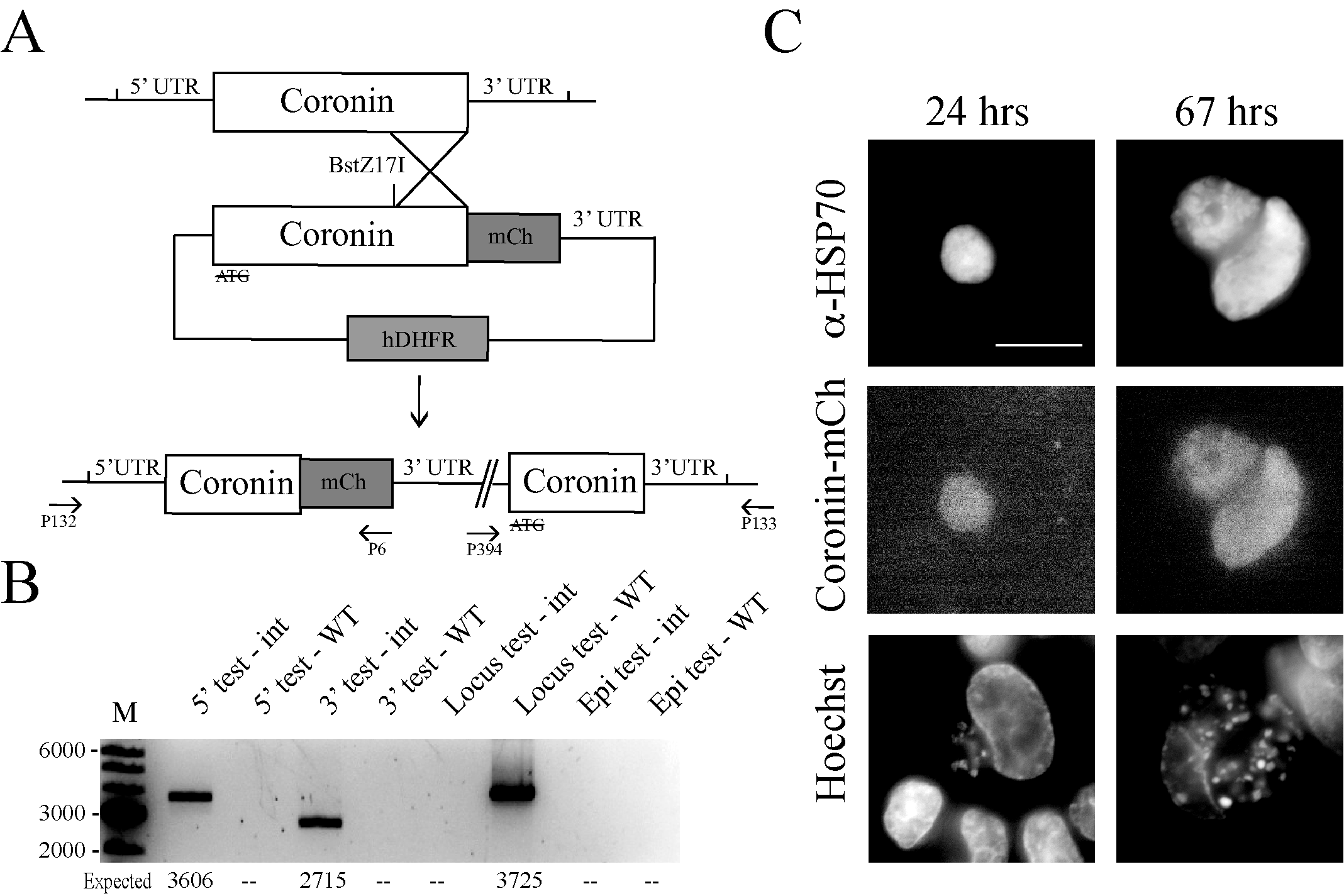

Supplement: S4 Fig — (A) Schematic of the strategy used for endogenous tagging of P. berghei coronin. A single crossover-strategy integrated a plasmid containing the resistance cassette (hDHFR) and the coronin C-terminal part fused to the coding sequence for mCherry after digestion of the plasmid with BstZ17I. Location of primers (S1 File) used for PCR in B are indicated. (B) Diagnositc PCR to confirm integration of the plasmid. Note that the PCR for the entire locus failed for the integrated plasmid, while a band was readily obtained for the WT locus. (C) Stills showing the expression of endogenous coronin-mCherry in HepG2 cells at 24 hours. and 67 hours post infection. The high background fluorescence in the coronin-mCherry channel indicates the relatively low expression level. HSP70 staining was used to identify liver stage parasites and Hoechst to reveal host cell and parasite DNA. Scale bar: 15 μm. (TIF) [file ppat.1005710.s004.tif]

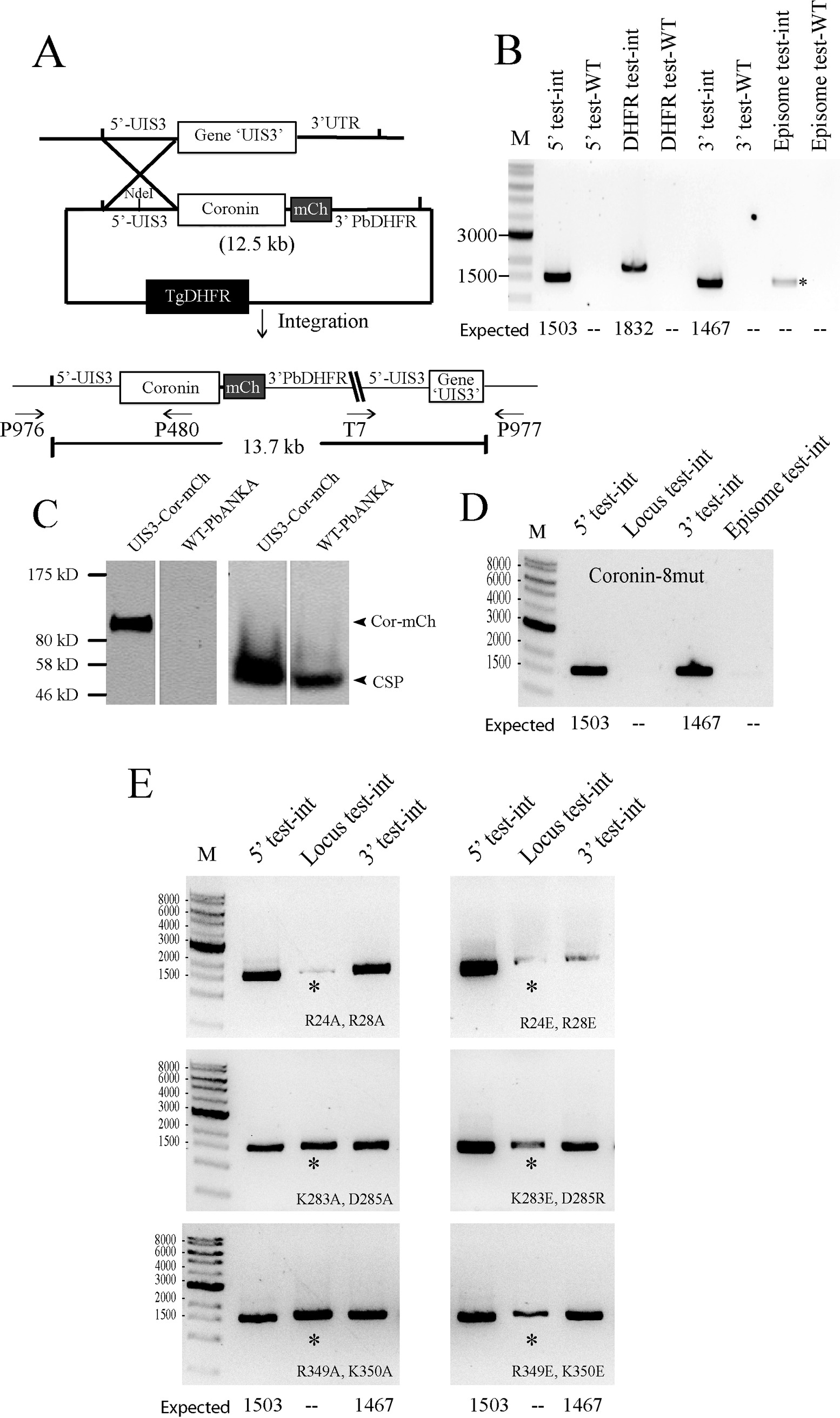

Supplement: S5 Fig — (A) Schematic of the strategy used to generate P. berghei lines expressing coronin-mCherry from the uis3 promoter. A single crossover-strategy integrated a plasmid containing the resistance cassette (hDHFR) and coronin fused to the coding sequence for mCherry after digestion of the plasmid with NdeI within the 5’UTR of uis3. Locations of primers (S1 File) used for PCR in B are indicated. (B) Genotyping PCR indicating desired integration of WT-coronin-mCherry in the UIS3 locus (5’ int, 3’ int and selection marker). Expected amplicon sizes are indicated below the gel. Band with * is non-specific due to non-clonality of the population. (C) Western blot showing expression of coronin-mCherry in salivary gland sporozoites probed with antibodies against mCherry (lane 1). The antibodies against circumsporozoite protein (CSP) were used as control (lane 3). CSP (lane 4) but not mCherry (lane 2) is detected in WT sporozoites. 105 sporozoites were loaded per lane. (D-E) Genotyping PCR showing proper integration of coronin mutants, Coronin-8mut (D) and R24A, R28A; R24E, R28E; K283A, D285A; K283E, D285R; R349A, K350A; R349E, K350E (E) (bands with * are non-specific due to the non-clonal population). Expected amplicon sizes are indicated below the gels. (TIF) [file ppat.1005710.s005.tif]

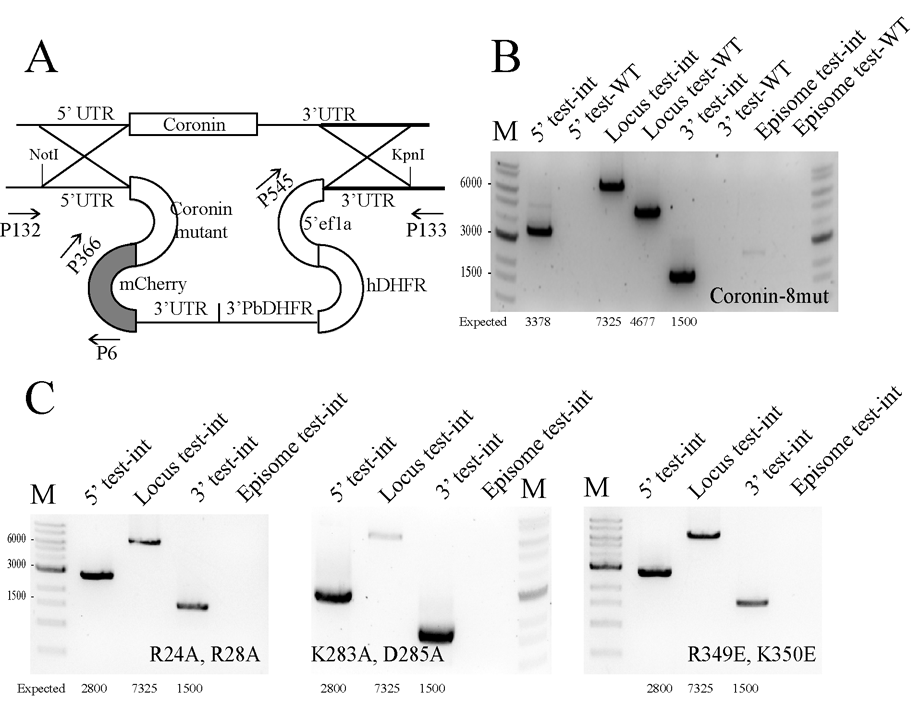

Supplement: S6 Fig — (A) Double homologous integration strategy for replacement of coronin mutants. Locations of primers (S1 File) used for PCR in B and restriction sites for generation of the linear DNA used for transfection are indicated. (B-C) Genotyping PCR confirming the replacement of endogenous coronin with coronin-8mut (B) and R24A, R28A; K283A, D285A; R349E, K350E (C). Expected amplicon sizes are indicated below the gels. (TIF) [file ppat.1005710.s006.tif]

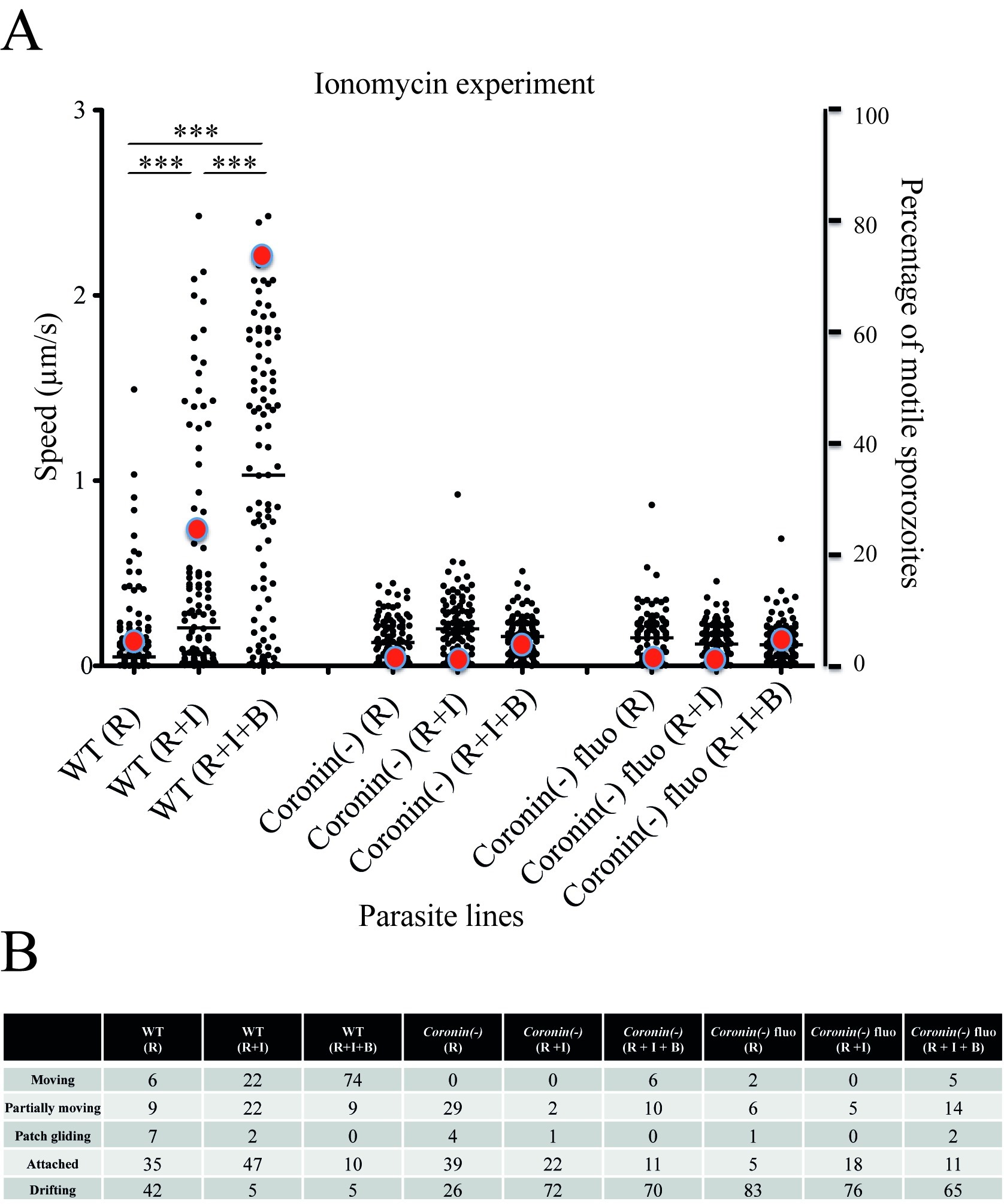

Supplement: S7 Fig — (A) Speed of sporozoites imaged in RPMI (R), RMPI supplemented with 100 nM ionomycin (R+I) and RMPI supplemented with 100 nM ionomycin and 3% bovine serum albumin (R+I+B). Note that while WT parasites move increasingly faster with ionomycin and ionomycin+BSA, there is no increase in motility in coronin(-) parasites. (B) Table listing the different movement patterns of WT and coronin(-) sporozoites incubated in RPMI (R), RPMI + 100nM ionomycin (R+I) and RPMI + 100nM ionomycin + 3% BSA (R+I+B). Note the increased numbers of motile parasites (red). (TIF) [file ppat.1005710.s007.tif]

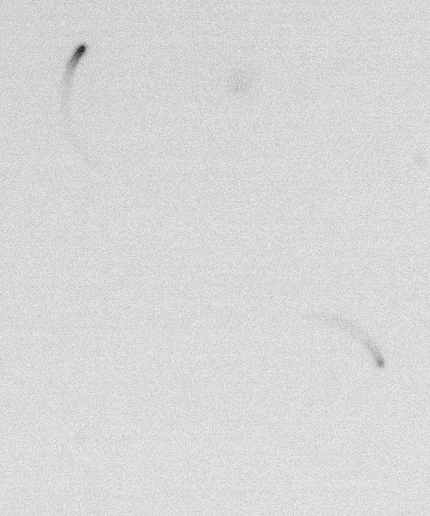

Supplement: S1 Movie — One image was taken every 3 seconds using a 63x (1.4 NA) objective. (GIF) [file ppat.1005710.s009.gif]
