# Supplementary material for: The Actin Filament-Binding Protein Coronin Regulates Motility in Plasmodium Sporozoites
Source: PLoS Pathog. 2016 Jul 13;12(7):e1005710. doi: 10.1371/journal.ppat.1005710 (PMC4943629; doi:10.1371/journal.ppat.1005710)
Supplement: S1 File — (DOCX) [file ppat.1005710.s008.docx]

**S7 information: Primers used in this study.**

|  |  |
| --- | --- |
| P124 | TCCCCGCGGGGCAATTTATAAGTAGAAATGG |
| P125 | ATTTGCGGCCGCGACAAACTTTAAATATTTTGTGACCC |
| P126 | CCCAAGCTTTTATTAATAGTAATATTTACATTAATATGTAGG |
| P127 | GGGGTACCCAAATCGAAAACACATATTCAC |
| P132 | CAACAATATCGTTATACATCAAGTTCG |
| P133 | CTTCAACTATGGTGTTATGTATTTCC |
| P177 | ATGCATAAACCGGTGTGTCTGG |
| P176 | CTAGACAGCCATCTCCATCTGG |
| P434 | TATGGATCCTTATTAATAGTAATATTTACATTAATATGTAGGAAGAAG |
| P435 | CGGGATATCGTTTCAAATCGAAAACACATATTCAC |
| P436 | AACCGGAATTCGTGAATGATGTCCCTTGTATCAAAAAC |
| P433 | TTCCATATGACCACCACCACCACCACCACCACCACCACCATTAGTTTTCGCAAATAATTTTTTACATGTTATAG |
| P6 | CGGGATCCTTACTTGTACAGCTCGTCCATGCCGCCGGTGG |
| P394 | TGTGCATTGAAGTACTTCG |
| P114 | CCCGCACGGACGAATCCAGATGG |
| P115 | CGCATTATATGAGTTCATTTTACACAATCC |
| P57 | GCACTAGTGCAGCAGCAGCAGTGAGCAAGGGCGAGGAGGATAACATGG |
| P58 | GCTTAATTAATTACTTGTACAGCTCGTCCATGCCGCC |
| L1 | GCCCGCGGACAATTTCATTTCGTTAGGGATCG |
| L2 | ATTTGCGGCCGCATACACTTTCATATATTTGTTATTTGTC |
| P71 | ATTTGCGGCCGCAAAATGGTGAATGATGTCCCTTGTATC |
| P72 | GCTCTAGAGCAGCAGCAGCAATTAGTTTTCGCAAATAATTTTTTACATG |
| P479 | GATGACTTGGCCATCTGCACGGCAATTACCGAATC |
| P480 | GATTCGGTAATTGCCGTGCAGATGGCCAAGTCATC |
| P481 | TATATATAATTGGTGCAGGGGCAGGGAATTGTAGAT |
| P482 | ATCTACAATTCCCTGCCCCTGCACCAATTATATATA |
| P483 | GTAATATATATAAATGTGCAATAGGAGCCATATATAAAAATGAAAATG |
| P484 | CATTTTCATTTTTATATATGGCTCCTATTGCACATTTATATATATTAC |
| P485 | CTATATCATTTTATGTACCAGCAGCAAATCCAAATATATTTCAG |
| P486 | CTGAAATATATTTGGATTTGCTGCTGGTACATAAAATGATATAG |
| P725 | GATGACTTGGAGATCTGCACGGAGATTACCGAATC |
| P726 | GATTCGGTAATCTCCGTGCAGATCTCCAAGTCATC |
| P727 | TATATATAATTGGTGAGGGGCGAGGGAATTGTAGAT |
| P728 | ATCTACAATTCCCTCGCCCCTCACCAATTATATATA |
| P729 | CTATATCATTTTATGTACCAGAAGAAAATCCAAATATATTTCAG |
| P730 | CTGAAATATATTTGGATTTTCTTCTGGTACATAAAATGATATAG |
| P976 | GGATATATGTATACATATATAATGAAAGAAAGATATGTCATGG |
| T7 | GAATACGACTCACTATAGGG |
| P977 | GTCCTGATGATAACAAAGCAATTGCAAC |
| P735 | CGAAGCTTAAATAATAATTATACTTTTGTATAAAGTAATGGAAATAATAATAATAAATTTGTCTTTTTAC |
| P736 | ATGGTACCGGGCATTGGTATTATTAAATATCTTCATGCATATTGTATATATATATAC |
| P563 | ATTTGCGGCCGCCCATATGATTTTTTTTAATTATATTATTTGTAATTTTTTATCTG |
| P564 | AACCGGAATTCTTTGGGTCACAAAATATTTAAAGTTTGTC |
| P366 | TCACCTTCAGCTTGGCG |
| P545 | GAAATATAAATAATTACGCCTAGTTAATAAAGGGCAC |
| GAPDH for | TGAGGCCGGTGCTGAGTATGTCG |
| GAPDH rev | CCACAGTCTTCTGGGTGGCAGTG |
| 18S rRNA for | AAGCATTAAATAAAGCGAATACATCCTTAC |
| 18S rRNA rev | GGAGATTGGTTTTGACGTTTATGTG |
